# Synthesis of the first Zn_6_-hexagon sandwich-tungstoantimonate *via* rearrangement of a non-lacunary Krebs-type polyoxotungstate†
†Dedicated to Professor Dr Dr h. c. Bernt Krebs on the occasion of his 80th birthday.
‡
‡Electronic supplementary information (ESI) available. CCDC 1576366, 1849600 and 1569737–1569739. For ESI and crystallographic data in CIF or other electronic format see DOI: 10.1039/c8dt02787k


**DOI:** 10.1039/c8dt02787k

**Published:** 2018-10-11

**Authors:** Elias Tanuhadi, Alexander Roller, Gerald Giester, Ioannis Kampatsikas, Annette Rompel

**Affiliations:** a Universität Wien , Fakultät für Chemie , Institut für Biophysikalische Chemie , Althanstraße 14 , 1090 Wien , Austria . Email: annette.rompel@univie.ac.at ; http://www.bpc.univie.ac.at; b Universität Wien , Fakultät für Chemie , Zentrum für Röntgenstrukturanalyse , Währinger Straße 42 , 1090 Wien , Austria; c Universität Wien , Fakultät für Geowissenschaften , Geographie und Astronomie , Institut für Mineralogie und Kristallographie , Althanstraße 14 , 1090 Wien , Austria

## Abstract

A novel synthetic pathway to obtain the first Zn_6_ hexagon tungstoantimonate [(Zn(H_2_O))_6_(*B*-α-SbW_9_O_33_)_2_]^6–^ ([Zn_6_-α-SbW_9_] (**1**)) *via* rearrangement of a non-lacunary Krebs-POM precursor (C_12_N_4_H_11_)_4_K_4_[(Mn(H_2_O)_3_)_2_((Mn_0.5_W_0.5_)O_2_)_2_(*B*-β-SbW_9_O_33_)_2_] ([Mn-β-SbW_9_]) has been developed. Addition of *ortho*-phenylenediamine (opda) in order to optimize the synthesis of [Mn-β-SbW_9_] led to the crystallization of a novel Krebs-type Zn-POM (C_12_N_4_H_11_)_4_Na_5_[((Zn_0_._8_W_0.2_)(H_2_O)_3_)_2_((Zn_0.2_W_0.8_)O_2_)_2_(*B*-β-SbW_9_O_33_)_2_] ([(Zn/W)_2_-β-SbW_9_] (**2**)) comprising four disordered Zn-centers after replacement of MnCl_2_ with ZnCl_2_. The compounds were characterized in the solid state by single-crystal and powder X-ray diffraction (XRD), IR spectroscopy, thermogravimetric analysis (TGA) and elemental analysis and in solution by UV-vis spectroscopy and ESI-mass spectrometry. The Krebs-POM archetype and the Zn_6_ hexagon tungstoantimonate have been investigated towards their interactions with Human Serum Albumin (HSA) as a model protein using SDS-PAGE and trypthophan fluorescence quenching.

## Abstract 2

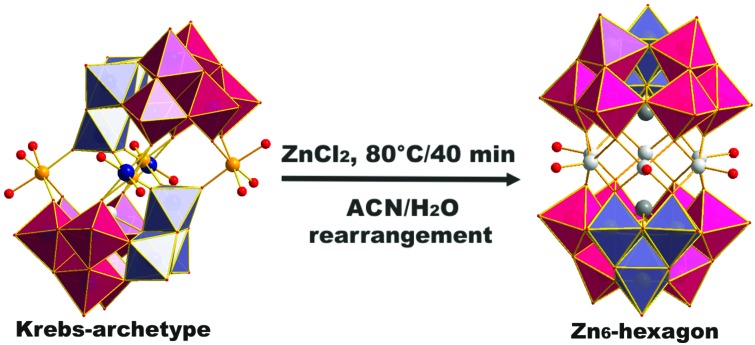
For the first time, the Krebs-POM archetype was used as a non-lacunary precursor expanding the rarely reported hexagon sandwich-POM family.

## 

Polyoxometalates (POMs)^1^ represent a large class of mostly anionic clusters, which are composed of metals in high oxidation states linked by oxygen atoms resulting in a vast variety of unique structures with possible applications in material science,^2^ photochemistry^3^ and biological chemistry^4^,^5^ including protein crystallography.^6^ Within the class of transition metal substituted POMs (TMSPs), the sandwich-type family represents the largest group. Sandwich-POMs generally are constructed by two lacunary species that are linked by heteroatoms. The family of sandwich POM compounds can be divided into further subtypes like the Krebs-7,8 and Hervé-9 type. Krebs-type POMs, which are constituted by two lone-pair containing β-Keggin lacunary fragments, *e.g.* [β-Sb(iii)W_9_O_33_]^9–^,^10^ have shown considerable importance in the field of both homo- and heterogeneous catalysis in the past.^11^ The first representatives of this structural family, [M_2_(H_2_O)_6_(WO_2_)_2_(β-SbW_9_O_33_)_2_]^(14–2*n*)–^ (M^*n*+^ = Fe^3+^, Co^2+^, Mn^2+^, Ni^2+^), were reported by Bösing *et al.* in 1997.^10^ Since then, a broad variety of Krebs-POM representatives has been reported, among them the tetra-substituted Krebs-polyanions, first synthesized and characterized by Kortz and co-workers in 2002.^8^ However, mixed transition metal substituted Krebs-type compounds remain rare. Most sandwich-type POMs contain two to five transition metals in the linking belt. Hexa-substituted analogues, however, especially the subclass of hexagon-sandwich polyoxotungstates, which comprise a hexagon-shaped linking-belt, have been reported scarcely, among them, Na_4_Zn_2_[Zn_2_(H_2_O)_10_(ZnCl)_6_(*B*-α-BiW_9_O_33_)_2_],^12^ [(Zn(Hen))_6_(*B*-α-AsW_9_O_33_)_2_]^13^ (en = ethylenediamine), [(CuCl)_6_(*B*-α-AsW_9_O_33_)_2_],^14^ [(MnCl)_6_(*B*-α-SbW_9_O_33_)_2_],^14^ [(CuCl)_6_(*B*-α-SbW_9_O_33_)_2_],^14^,^15^ and [(MnCl)_6_(*B*-α-AsW_9_O_33_)_2_].^15^ To the best of our knowledge, [(MnCl)_6_(*B*-α-SbW_9_O_33_)_2_] and [(CuCl)_6_(*B*-α-SbW_9_O_33_)_2_], published by Yamase *et al.*^14^,^15^ represent the only hexa-substituted sandwich POMs containing the [SbW_9_O_33_]^9–^ building block.

The majority of POM compounds is prepared by a simple one-pot synthetic procedure, which follows mainly self-assembly mechanisms. These mechanisms are rather complex and most often lead to novel, yet unprecedented products, making the selective preparation of new hexagon sandwich POM com-pounds challenging. Therefore, the development of synthetic routes to design the desired clusters is of special interest. Herein, we report a novel multistep-synthesis, which includes the rearrangement of a Krebs-type manganese POM, [(Mn(H_2_O)_3_)_2_((Mn_0.5_W_0.5_)O_2_)_2_(*B*-β-SbW_9_O_33_)_2_] abbreviated [Mn-β-SbW_9_], in order to obtain the first Zn_6_-hexagon tungsto-antimonate [(Zn(H_2_O))_6_(*B*-α-SbW_9_O_33_)_2_]^6–^ in the following termed [Zn_6_-α-SbW_9_] (**1**). On the way to synthesizing the target compound, we were able to obtain the literature known [Mn-β-SbW_9_] POM cluster more selectively and faster^16^ by using *o*-phenylenediamine (opda). In the presence of Zn^2+^ applying this optimization strategy selectively led to the crystallization of the novel Krebs-type zinc compound (C_12_N_4_H_11_)_4_Na_5_[((Zn_0.8_W_0.2_)(H_2_O)_3_)_2_((Zn_0.2_W_0.8_)O_2_)_2_(*B*-β-SbW_9_O_33_)_2_] ([(Zn/W)_2_-β-SbW_9_] (**2**)) composed of a linking belt with four disordered zinc centres. Replacement of Zn^2+^ with Cd^2+^ and decrease in pH yielded the novel mixed Krebs-type compound (TMA)_2_Na_6_H_8_[(Mn(H_2_O)_3_)_2_(CdCl_0.8_O_1.2_)_2_(*B*-β-SbW_9_O_33_)_2_] ([Cd_2_Mn_2_-β-SbW_9_] (**3**)), TMA = tetramethylammonium. As the interaction of Krebs-type POTs with proteins has never been investigated, the interaction of all three Krebs-type clusters [Mn-β-SbW_9_], [(Zn/W)_2_-β-SbW_9_] (**2**) and [Cd_2_Mn_2_-β-SbW_9_] (**3**) and the Zn_6_-hexagon sandwich-tungstoantimonate [Zn_6_-α-SbW_9_] (**1**) with Human Serum Albumin (HSA) as a model protein has been studied by sodium dodecyl sulfate-polyacrylamide gel electrophoresis (SDS-PAGE) analysis and tryptophan fluorescence quenching.

The first step in the synthesis of [Zn_6_-α-SbW_9_] (**1**) (Fig. 1) was the preparation of the Krebs-POM precursor [Mn-β-SbW_9_].^16^ Upon addition of opda to a stirred reaction mixture of Na_9_[*B*-SbW_9_O_33_] and MnCl_2_, the *in situ* formed [(Mn(H_2_O)_3_)_2_((Mn_0.5_W_0.5_)O_2_)_2_(*B*-β-SbW_9_O_33_)_2_] catalyzed the oxidation reaction of opda to 2,3-diaminophenazine (2,3-DAP) (Scheme 1), which co-crystallized together with [Mn-β-SbW_9_] (Fig. S9‡). Addition of opda resulted in a significantly lower water solubility of [Mn-β-SbW_9_], which subsequently facilitated its crystallization process. X-ray-crystallographic studies on [Mn-β-SbW_9_] (CCDC number ; 1569737, Tables S6 and S7‡) revealed π–π-interactions between the aromatic 2,3-DAP systems. As a result, a more stabilized crystal packing was obtained (Fig. S9‡), which in turn led to an accelerated crystallization of [Mn-β-SbW_9_] upon cooling of the stirred solution to room temperature. It is worth noting that literature known synthetic protocols of [Mn-β-SbW_9_] lead to a mixture of the anions [Na_3_(H_2_O)_6_Mn_3_(μ-OAc)_2_(*B*-α-SbW_9_O_33_)_2_]^11–^ and [Mn_3_W(H_2_O)_8_(*B*-β-SbW_9_O_33_)_2_]^4–^,^17^ whereas addition of opda selectively enables isolation of the Krebs-β-SbW_9_ anion (80% yield based on W). Besides SXRD, [Mn-β-SbW_9_] was characterized in the solid state by ATR-IR spectroscopy (Fig. S1‡). The same reaction procedure using ZnCl_2_ instead of MnCl_2_ led to the crystallization of the novel Krebs-type zinc compound [(Zn/W)_2_-β-SbW_9_] (**2**) with four zinc centres disordered with tungsten (CCDC number ; 1569739, Tables S8 and S9‡). However, the full d^10^-electron configuration of Zn^2+^ led to only little opda-conversion, resulting in a lower product yield of [(Zn/W)_2_-β-SbW_9_] (**2**) (12% yield based on W). As a matter of fact, addition of catalytic amounts of FeCl_3_,^18^ to overcome this problem, led to a higher opda-conversion resulting in an optimized yield of [(Zn/W)_2_-β-SbW_9_] (**2**) (70% yield based on W). Interestingly, omission of opda in the procedure leads to [Zn_2_(H_2_O)_6_(WO_2_)_2_(*B*-β-SbW_9_O_33_)_2_]^10^–^19^ not exhibiting (Zn/W) disorders as characterized by SXRD, ATR-IR spectroscopy (Fig. S2‡) and elemental analysis.

**Fig. 1:**
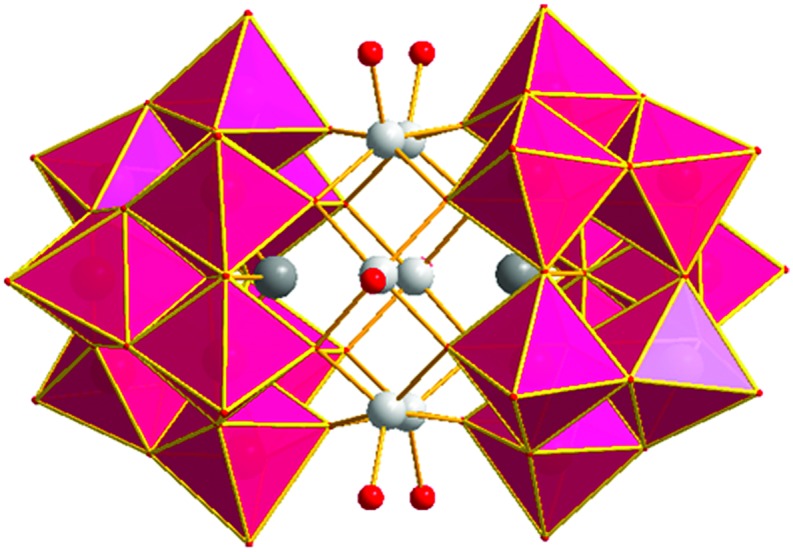
Polyhedral representation of [Zn_6_-α-SbW_9_] (**1**). Colour legend: WO_6_, pink; Sb, dark grey; Zn, silver; O_t_, red.

**Scheme 1:**
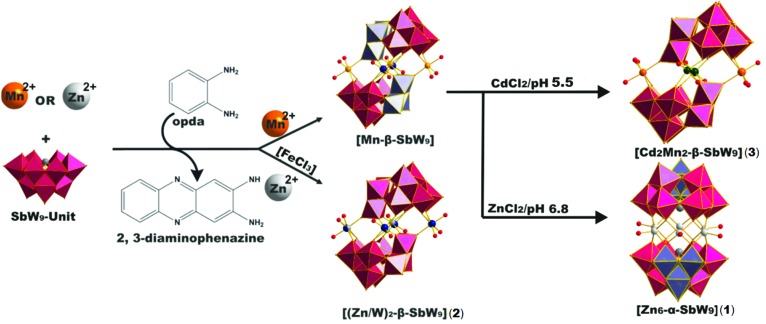
Structure and synthesis of [Zn_6_-α-SbW_9_] (**1**) and [(Zn/W)_2_-β-SbW_9_] (**2**). The synthesis starts from the [SbW_9_] unit and the transition metal (TM) of choice. [Mn-β-SbW_9_] was used as precursor for the synthesis of the title compound, which was obtained by exchange of the manganese linker cations with zinc cations and β/α-isomerization of the precursor. Counter cations are omitted for clarity. Color legend: WO_6_, pink octahedra; 60° rotated WO_6_, ice blue octahedra; Sb, dark grey spheres; O, dark red spheres; Zn, light grey spheres; Mn, orange spheres; Cd, dark green spheres, W in the linking belt, dark blue spheres. Opda – *o*-phenylenediamine; 2,3-DAP – 2,3-diaminophenazine, ACN – acetonitrile.

Attempts to prepare [Zn_6_-α-SbW_9_] (**1**) by reacting Na_9_[*B*-α-SbW_9_O_33_] with ZnCl_2_ were not successful. Considering the synthesis of [(CuCl)_6_(*B*-α-AsW_9_O_33_)_2_]^12–^, which only worked by preparing the non-lacunary [{EuH_2_O}_2_(*B*-α-AsW_9_O_33_)_2_]^12–^ polyanion to replace the europium-linker cations in the linking belt with a Cu_6_-hexagon in a subsequent reaction step, we decided to use the Krebs-POM [Mn-β-SbW_9_] as a non-lacunary precursor to prepare the Zn_6_-hexagon sandwich target compound [Zn_6_-α-SbW_9_] (**1**). Regarding the decreased water solubility of [Mn-β-SbW_9_], the second reaction step (Scheme 1) was performed by using a solvent mixture of acetonitrile : water = (1 : 4). After addition of an excess of ZnCl_2_ to the dissolved [Mn-β-SbW_9_] precursor, the reaction solution was refluxed for 40 min at pH 6.8. An aqueous solution of SbW_9_ contains alpha and beta species in equilibrium. Based on SXRD studies performed by Krebs and co-workers^10^ on isolated SbW_9_ containing species at different pH values, the stable pH range of the [α-SbW_9_]-moiety is known to be between 6.0 and 7.5. Lower pH values result in a higher tendency of the SbW_9_ unit to undergo further condensation. This increased tendency further results in the generation of the [β-SbW_9_]-isomer, which proves to be stable in the pH range of 4.5 to 5.5 indicated by the formation of the literature known Zn_2_-β-SbW_9_-Krebs-type analogue^19^ at pH values lower than 6.0 and formation of [Cd_2_Mn_2_-β-SbW_9_] (**3**) upon replacing ZnCl_2_ with CdCl_2_ at pH 5.5 (Scheme 1). Filtration of the brownish reaction mixture, after dropwise addition of a tetramethylammonium chloride (TMACl) solution, gave a yellowish solution, which was left to evaporate for one week to give a mixture of red crystal plates (TMA)(C_12_N_4_H_11_)_5_[(Zn(H_2_O))_6_(*B*-α-SbW_9_O_33_)_2_] ((TMA)(C_12_N_4_H_11_)_5_[Zn_6_-α-SbW_9_] (**1**)) (CCDC ; 1569738, Tables S10 and S11‡) and colourless block shaped crystals (TMA)_6_[(Zn(H_2_O))_6_(*B*-α-SbW_9_O_33_)_2_] ((TMA)_6_[Zn_6_-α-SbW_9_] (**1**)) (CCDC ; 1576366, Tables S12 and S13.‡ Replacement of zinc chloride with cadmium chloride at pH 6.8 to yield the cadmium containing hexagon analogue, resulted in precipitates, which despite all our efforts could not be identified.

Interestingly, XRD studies of both crystal fractions revealed the same Zn_6_-hexagon anion in both crystal fractions, with the 2,3-DAP cation lacking in the colourless block shaped crystals, which indicates the complete rearrangement of the Krebs-type precursor [Mn-β-SbW_9_] to [Zn_6_-α-SbW_9_] (**1**). By using the inorganic di-substituted counterpart Na_10_[Mn_2_(H_2_O)_6_(WO_2_)_2_(*B*-β-SbW_9_O_33_)_2_]·40H_2_O^19^ as a precursor, (TMA)_6_[Zn_6_-α-SbW_9_] (**1**) could be obtained as a pure crystalline phase in lower yields (12%). The same reaction procedure, replacing ZnCl_2_ with CdCl_2_ in an acetate buffer (pH = 5.5) led to the formerly mentioned dominance of the beta isomer resulting in the novel mixed substituted Krebs-POM compound [Cd_2_Mn_2_-β-SbW_9_] (**3**) (CCDC ; 1849600, Tables S14 and S15‡) (Scheme 1).

(TMA)_6_[Zn_6_-α-SbW_9_] (**1**) was additionally characterized in the solid state by ATR-IR spectroscopy (Fig. S3 and S4‡), PXRD (Fig. S13‡) and elemental analysis. SXRD-analysis showed that the four polyanions, [Mn-β-SbW_9_], [(Zn/W)_2_-β-SbW_9_] (**2**), [Cd_2_Mn_2_-β-SbW_9_] (**3**) and (TMA)(C_12_N_4_H_11_)_5_[Zn_6_-α-SbW_9_] (**1**), crystallize in a triclinic space group *P*1[combining macron], whereas (TMA)_6_[Zn_6_-α-SbW_9_] (**1**) belongs to the monoclinic space group *C*_2/*m*_.

The crystal structure of [(Zn/W)_2_-β-SbW_9_] (**2**) shows two [β-SbW_9_]-lacunary species linked by four zinc centres. All four zinc centres exhibit a disorder with tungsten resulting in the typical Krebs-type structure with idealized *C*_2h_ symmetry (Fig. S10‡). Regarding the synthetic conditions of [(Zn/W)_2_-β-SbW_9_] (**2**), which include the use of an acidic buffer (pH = 4.8), the disorder with tungsten is in accordance with the results for the disordered alpha-arsenotungstate compounds observed at lower pH values, reported by Kortz *et al.* in 2001.^20^ Zinc and tungsten show inverse occupancy ratios at the inner (Zn : W = 20 : 80) and outer (Zn : W = 80 : 20) site of the linking belt. The zinc centres exhibit a distorted octahedral coordination environment with three H_2_O ligands bound to the peripheral zinc positions and Zn–O bond lengths ranging from 1.664(2) at the inner site of the belt to 2.237(1) Å between the peripheral zinc centres and the H_2_O ligands (Fig. S11‡). As for the architecture of [Cd_2_Mn_2_-β-SbW_9_] (**3**), SXRD studies revealed two [β-SbW_9_]-lacunary species linked by a belt comprising two Cd^2+^ centres at the inner positions, whereas the peripheral sites of the belt are occupied by two Mn^2+^ metal centres. All four metal centres exhibit a distorted octahedral coordination environment with Cd–O bond lengths ranging from 1.962(1) to 2.105(2) Å and Mn–(OH_2_) distances between 2.164(1) and 2.209(2) Å. The crystal structure of [Zn_6_-α-SbW_9_] (**1**), (Fig. 1) presents a polyanion with idealized *D*_3d_ symmetry consisting of two [(Zn(H_2_O))_3_(*B*-β-SbW_9_O_33_)] building blocks related by an inversion centre (Fig. S11A‡). Both building blocks are formed by rearrangement of the precursor [β-SbW_9_] moiety to the alpha isomer upon 60° rotation of a W_3_O_13_ unit. Occupation of the three vacant sites with the corresponding number of Zn^2+^ results in the [(Zn(H_2_O))_3_(*B*-α-SbW_9_O_33_)] building block comprising three zinc centres arranged in a triangular shape with Zn–Zn distances ranging from 3.089 to 3.114 Å and a bond angle of 119.49°. Each zinc ion inside the belt shows distorted square-pyramidal coordination geometry with four oxygen atoms belonging to two [SbW_9_O_33_] clusters and a terminal H_2_O-ligand (O_t_) (Fig. S11B‡). The Zn–O distances vary between 2.025 and 2.062 Å with O_t_–Zn–O bond angles from 102.061° to 109.783°.

The number of water molecules in the novel compounds [(Zn/W)_2_-β-SbW_9_] (**2**)·13H_2_O, [Cd_2_Mn_2_-β-SbW_9_] (**3**)·51.5H_2_O and (TMA)_6_[(Zn(H_2_O))_6_(B-α-SbW_9_O_33_)_2_] (**1**)·37.5H_2_O was determined using thermogravimetric analysis (TGA) (Fig. S6–S8 and Tables S2–S4‡). The five polyanions were investigated by UV-vis spectroscopy (Fig. S15 and Table S16‡). The spectra are characterized by an absorption maximum at 272 nm attributed to the pπ(O_b_) → dπ*(W) ligand-to-metal charge-transfer typical for the Keggin-type framework.^21^ A second absorption maximum in the spectra of the 2,3-DAP-containing polyanions^22^ was observed around 425 nm corresponding to aromatic transitions. ESI-MS was performed on (TMA)(C_12_N_4_H_11_)_5_[(Zn(H_2_O))_6_(*B*-α-SbW_9_O_33_)_2_] (**1**). The major peaks observed show the [(Zn(H_2_O))_6_(*B*-α-SbW_9_O_33_)_2_]^6–^ moiety and a series of various tungsten related species based on the polyanion (Fig. S16 and Table S17‡).

The interactions of the peripheral Lewis acid metal centres in the compounds [Mn-β-SbW_9_], [(Zn/W)_2_-β-SbW_9_] (**2**), [Cd_2_Mn_2_-β-SbW_9_] (**3**) and (TMA)_6_[(Zn(H_2_O))_6_(*B*-α-SbW_9_O_33_)_2_] (**1**) with Human Serum Albumin (HSA) as a model protein were investigated to assess whether sandwich POM compounds show any proteolytic character in solution(s) with proteins, as has been reported for other POM archetypes.^23^ SDS-PAGE (Fig. S17‡) was applied in 10 mM NaOAc buffer pH 5.5 (Fig. S17G–N,‡ for [Mn-β-SbW_9_], (**2**), (**1**) and (**3**)) and in 25% DMSO solution (Fig. S17A–F,‡ for [Mn-β-SbW_9_], (**2**) and (**3**)) respectively, to ensure a relaxed conformation of the model protein. The results reveal that all four compounds show no hydrolytic activity as indicated by intact HSA bands at 66 kDa even at 65 °C and 100-fold excess of each sandwich POM.

Fluorescence quenching as a well-established method to investigate ligand–protein interactions has been applied to investigate the interactions of HSA/BSA and different POM archetypes, including the Keggin,^24^ Wells-Dawson^25^ and wheel-shape-structured^26^ POM architectures. Herein, fluorescence quenching was applied to gain more insight into the interaction between the sandwich POM archetype and the fluorophore tryptophan of HSA for the first time. All four fluorescence spectra of HSA show a maximum at 313 nm, which decreases with increased POM concentration indicating the binding of POM to HSA (Fig. S18–S21‡). Furthermore, a clear hypsochromic shift of the maximum to lower wavelengths upon increased POM concentration indicates a decrease in polarity within the immediate tryptophan environment. It is worth noting that all POMs form a 1 : 1 complex with the protein, except for [Cd_2_Mn_2_-β-SbW_9_] (**3**), which showed the strongest interaction with HSA resulting in 1 to 2 POM molecules binding to the protein (Table S18‡). This could be explained both by electrostatic and structural factors, as [(Mn(H_2_O)_3_)_2_(CdCl_0.8_O_1.2_)_2_(*B*-β-SbW_9_O_33_)_2_]^16–^ [Cd_2_Mn_2_-β-SbW_9_] (**3**) shows the highest negative charge relative to the other Krebs-POMs [(Mn(H_2_O)_3_)_2_((Mn_0.5_W_0.5_)O_2_)_2_(*B*-β-SbW_9_O_33_)_2_]^8–^ ([Mn-β-SbW_9_]) and [((Zn_0.8_W_0.2_)(H_2_O)_3_)_2_((Zn_0.2_W_0.8_)O_2_)_2_(*B*-β-SbW_9_O_33_)_2_]^9–^ ([(Zn/W)_2_-β-SbW_9_] (**2**)), whereas the title compound [(Zn(H_2_O))_6_(*B*-α-SbW_9_O_33_)_2_]^6–^ ([Zn_6_-α-SbW_9_] (**1**)), which shows a stronger binding compared to [(Mn(H_2_O)_3_)_2_((Mn_0.5_W_0.5_)O_2_)_2_(*B*-β-SbW_9_O_33_)_2_]^8–^ ([Mn-β-SbW_9_]), comprises more accessible metal centres. These results suggest non-proteolytic POM protein interactions of the sandwich-POM archetype, which, potentially could be used as an additive in POM assisted protein crystallography.^6^

## Conclusions

In conclusion, the synthetic pathway presented in this paper may open novel reaction routes for the synthesis of new hexagon-POM structures. The potential of the Krebs-POM archetype as a non-lacunary synthetic precursor and its interactions with HSA as a model protein have been explored.

## Conflicts of interest

There are no conflicts to declare.

## Supplementary Material

Supplementary information

Crystal structure data
