# Supplementary material for: Synthesis of the first Zn6-hexagon sandwich-tungstoantimonate via rearrangement of a non-lacunary Krebs-type polyoxotungstate
Source: Dalton Trans. 2018 Oct 11;47(44):15651–5. doi: 10.1039/c8dt02787k (PMC6256361; doi:10.1039/c8dt02787k)
Supplement: Supplementary file 1 [file DT-047-C8DT02787K-s001.pdf]

Electronic Supplementary Material (ESI) for Dalton Transactions.
This journal is © The Royal Society of Chemistry 2018

ELECTRONIC SUPPLEMENTARY INFORMATION

Synthesis of the first Zn_6-hexagon sandwich-tungstoantimonate via rearrangement of a non-lacunary Krebs-type polyoxotungstate

Elias Tanuhadi[†], Alexander Roller[‡], Gerald Giester[§], Ioannis Kampatsikas[†] and Annette Rompel[*,†]

[*]correspondence to annette.rompel@univie.ac.at

[†]Universität Wien, Fakultät für Chemie, Institut für Biophysikalische Chemie, Althanstraße 14, 1090 Wien, Austria. www.bpc.univie.ac.at

[‡]Universität Wien, Fakultät für Chemie, Zentrum für Röntgenstrukturanalyse, Währinger Straße 42, 1090 Wien, Austria.

[§] Universität Wien, Fakultät für Geowissenschaften, Geographie und Astronomie, Institut für Mineralogie und Kristallographie, Althanstraße 14, 1090 Wien, Austria

Content

1. General Information 3

2. Experimental Procedure 5

2.1. Preparation of $(C_{12}N_4H_{11})_4K_4[(Mn(H_2O)_3)_2(Mn_{0.5}W_{0.5}O_{0.5})_2(B\text{-}\beta\text{-}SbW_9O_{33})_2]$ ([Mn-β-SbW_9]) 5

2.2. Preparation of $(C_{12}N_4H_{11})_4Na_5[(Zn_{0.8}W_{0.2}(H_2O)_3)_2(Zn_{0.2}W_{0.8}O_2)_2(B\text{-}\beta\text{-}SbW_9O_{33})_2]$ ([$(Zn/W)_2$-β-SbW_9]) (**2**) 5

2.3. Preparation of $[(Zn(H_2O))_6(B\text{-}\alpha\text{-}SbW_9O_{33})_2]^{6-}$ ([Zn_6-α-SbW_9]) (**1**) 5

2.4. Preparation of $(TMA)_2Na_6H_8[(Mn(H_2O)_3)_2(CdCl_{0.8}O_{1.2})_2(B\text{-}\beta\text{-}SbW_9O_{33})_2]$ [Cd_2Mn_2-β-SbW_9] (**3**) 6

3. IR-spectra 7

4. Thermogravimetric Analysis 12

5. Single-Crystal X-ray Diffraction 14

6 PXRD 22

7 UV-vis Spectroscopy 25

8 ESI-Mass Spectrometry 26

9 POM-Protein Interactions 27

10 References 33

1. General Information

All reagents and chemicals were of high-purity grade and were used as purchased without further purification. $Na_9[B\text{-}\alpha\text{-}SbW_9O_{33}]$ was prepared according to the literature procedure reported by Bösing *et al.*.[1]

Attenuated total reflection Fourier−transform Infrared Spectroscopy: All spectra were recorded on a Bruker Tensor 27 IR Spectrometer equipped with a single−reflection diamond−ATR unit. Frequencies are given in cm^{-1}, intensities denoted as w = weak, m = medium, s = strong.

Thermogravimetric analysis: TGA was performed on a Mettler SDTA851e Thermogravimetric Analyzer under nitrogen flow with a heating rate of 5 K min^{-1} in the region 298−1023 K.

UV–Vis spectroscopy: UV−Vis spectra were collected on a Shimadzu UV 1800 spectrophotometer. The spectra were recorded in 50 % (v/v) DMSO solution.

Electrospray Ionization Mass Spectrometry: The title compound $[Zn_6\text{-}\alpha\text{-}SbW_9]$ (**1**) was investigated with an ESI−Qq−oaRTOF supplied by Bruker Daltonics Ltd. at the Massenspektrometriezentrum, University of Vienna. Bruker Daltonics Data Analysis software was used to analyze the results. The analysis was carried out in 1:1 mixture of water/ACN, collected in negative ion mode and with the spectrometer calibrated with the standard tune−mix to give a precision of ca. 5 ppm in the region of m/z 300−3000.

Elemental analysis: Elemental analysis was performed at the Institute of Chemical Technologies and Analytics, Technische Universität Wien.

An iCAP 6500 series ICP-OES spectrometer (Thermo Scientific, USA) has been used for simultaneous multielement analysis. The ICP-OES was equipped with a standard sample introduction system consisting of a concentric nebulizer and a cyclonic spray chamber. Transportation of sample solutions was performed by the peristaltic pump of the iCAP 6500 coupled to an ASX-520 auto sampler (Cetac, USA). The conditions used for determination of background corrected emission signals are presented in Table 1. Per element two sensitive and non interfered emission lines were used, the first line for measurement, the second line for quality control.

Table S1: Instrumental parameters and analytical wavelengths [nm]

Parameter	Value	Parameter	Value
Rf power	1200 W	Nebulizer flow	0.8 l min^{-1}
Sample flow	0.80 ml min^{-1}	Auxiliary flow	1.0 l min^{-1}
Read delay	70 s	Coolant flow	15 l min^{-1}
Observation height	12 mm		
Integration time	10 s		
Number of replicates	5		
Background	constant shift from analytical line		

Element	Wavelength I [nm]	Wavelength II [nm]
Mn	257.610	259.373
Sb	206.833	231.147
W	239.709	209.475
Zn	213.856	202.548
In	230.606	

Prior to ICP-OES measurement clear sample solutions were diluted 1:10 with 1%-HNO_3 (vol/vol), thereby Indium with a concentration level of 5 mg/L has been added as an internal standard to all sample solutions. Blank solutions were prepared in the same manner with the use of high purity water instead of sample solution. Quantification of derived emission signals was based on an external calibration function determined with aqueous standard solutions using Indium as internal standard.

Chemicals and Reagents

All reagents and standard solutions used for the determination of the investigated elements were of adequate quality. High purity water was obtained by purifying de-ionized water (reverse osmosis/ion exchange combination Euro 20 plus, SG Water Systems, Germany) with an Easypure 2 system (Thermo Barnstead, USA). Nitric acid was of p.a. grade purity (Merck, Darmstadt, Germany). A certified stock solution of Indium (TraceCert ICP standard) was purchased from Fluka (Germany). Certified stock solutions (1000 mg L^{-1}) of Mn, Sb, W and Zn were procured from Merck (Darmstadt, Germany) and used for the preparation of calibration standards. Appropriate working solutions were prepared by dilution of the stock solutions with 1.0 % nitric acid (v/v) just before use.

SDS–PAGE was performed according to a previously published procedure[2] using Precision Plus Protein Standard Dual Color (Bio−Rad) as molecular weight marker. Samples were applied to 14 % polyacrylamide gels mixed with reduced loading dye. Sample load onto the gel was 5 µg. Gels were stained with Coomassie Brilliant Blue. Imaging of the gels was applied with Gel Doc™ XR of BIO−RAD.

Tryptophan fluorescence spectroscopy: [Mn-β-SbW_9], [$(Zn/W)_2$-β-SbW_9],] (**2**), [Zn_6-α-SbW_9] (**1**) and [Cd_2Mn_2-β-SbW_9] (**3**) were recorded on a Infinite 200 microplate reader using Nunclon 96 Flat Bottom Black Polysterol plates. The range of the emission wavelength was between 290 and 450 nm. The absorbance from the POMs was deducted from raw quenching data by applying equation **1**.

$$F_{corr} = F_{obs} * antilog\left(\frac{0.605 * (OD_{ex} + OD_{em})}{2}\right) \quad (1)$$

where F_{corr} and F_{obs} are the fluorescence intensities after and before correcting inner filter effects, respectively, OD_{ex} the optical density at the excitation wavelength and OD_{em} the optical density at emission wavelength.

The further evaluation of the results was done with a derived Stern-Volmer equation **2**[3].

$$\log\left[\frac{F_0 - F_{corr}}{F_{corr}}\right] = logK_q + n * log[Q] \qquad (2)$$

where F_0 and F_{corr} are the fluorescence intensities in the absence and presence of the quencher, respectively, K_q the quenching rate constant of the protein, n the number of bound molecules and [Q] the concentration of the quencher.

2. Experimental Procedure

2.1. Preparation of $(C_{12}N_4H_{11})_4K_4[(Mn(H_2O)_3)_2(Mn_{0.5}W_{0.5}O_{0.5})_2(B\text{-}\beta\text{-}SbW_9O_{33})_2]$ ([Mn-β-SbW_9])

To a stirred solution of $Na_9[B\text{-}\alpha\text{-}SbW_9O_{33}]$ (1.07g, 0.35 mmol) in aqueous sodium acetate buffer (100 ml, pH 4.8, NaOAc/AcOH 0.5 M), manganese chloride $MnCl_2 \cdot 4\ H_2O$ (29.69 mg, 0.15 mmol) was added. The resulting yellow reaction mixture was stirred at 70 °C for 10 minutes. Ortho-phenylenediamine (540.7 mg, 5 mmol) was added to the reaction solution and the mixture was stirred for further 3 h at 85 °C. A significant change from yellow to dark orange over the time period of 3 h was noticed. KCl (10 ml, 0.5 M) was added dropwise to the hot reaction mixture. Dark red crystal plates of [Mn-β-SbW_9] were obtained upon cooling the reaction mixture to room temperature and further evaporation at 18°C gave a total yield of 80% based on W of the compound after 3 days.

2.2. Preparation of $(C_{12}N_4H_{11})_4Na_5[(Zn_{0.8}W_{0.2}(H_2O)_3)_2(Zn_{0.2}W_{0.8}O_2)_2(B\text{-}\beta\text{-}SbW_9O_{33})_2]$ ([(Zn/W)$_2$-β-SbW_9]) (**2**)

$Na_9[B\text{-}\alpha\text{-}SbW_9O_{33}]$ (1.07 g, 0.35 mmol) and $ZnCl_2$ (204.45 mg, 1.5 mmol) were dissolved in sodium acetate buffer (100 ml, pH 4.8, NaOAc/AcOH 0.5 M). The resulting reaction mixture was refluxed at 80 °C for 30 min. To the colorless solution, *o*-phenylenediamine (216.28 mg, 2 mmol) was added. The mixture was stirred for 1.5 h at 85 °C. A color change from colorless to yellow was observed. After 1.5 h, $FeCl_3$ (32.4 mg, 0.2 mmol) was added and the solution was refluxed at 90 °C for further 1.5 h. A dark orange reaction mixture was obtained. The solution was filtered and cooled to room temperature. Evaporation at 18 °C gave dark red crystal plates of [(Zn/W)$_2$-β-SbW_9] (**2**) after 3 days (yield 70 % based on W). Elem. anal. Calcd (found): Zn, 2.0 (2.4); Sb, 3.7 (4.1); W, 55.6 (55.5).

2.3. Preparation of $[(Zn(H_2O))_6(B\text{-}\alpha\text{-}SbW_9O_{33})_2]^{6-}$ ([Zn_6-α-SbW_9]) (**1**)

[Mn-β-SbW_9] (193 mg, 0.034 mmol) was dissolved in 15 ml of acetonitrile/water mixture (1:4). The pH of the resulting solution was adjusted to 6.8 by dropwise addition of 0.1 M NaOH. $ZnCl_2$ (29.8 mg, 0.218 mmol) was added and the reaction mixture was heated to 80 °C for 2

h. A solution of tetramethylammonium chloride (TMACl) (3 ml, [0.4 M]) was added dropwise to the hot reaction mixture, which was filtered after cooling to room temperature. Evaporation of the resulting light yellowish solution at 18°C gave a mixture of block shaped colorless crystals of $(TMA)_6[(Zn(H_2O))_6(B\text{-}\alpha\text{-}SbW_9O_{33})_2]$ ($(TMA)_6[Zn_6\text{-}\alpha\text{-}SbW_9]$) ((yield 24 % based on W) Elem. anal. Calcd (found): Zn, 6.5 (7.2); Sb, 4.0 (4.0); W, 54.4 (54.7) and red crystal plates of $(TMA)(C_{12}N_4H_{11})_5[(Zn(H_2O))_6(B\text{-}\alpha\text{-}SbW_9O_{33})_2]$ ($(TMA)(C_{12}N_4H_{11})_5[Zn_6\text{-}\alpha\text{-}SbW_9]$) (yield 10 % based on W) after one week.

2.4. Preparation of $(TMA)_2Na_6H_8[(Mn(H_2O)_3)_2(CdCl_{0.8}O_{1.2})_2(B\text{-}\beta\text{-}SbW_9O_{33})_2]$ $[Cd_2Mn_2\text{-}\beta\text{-}SbW_9]$ (**3**)

$[Mn\text{-}\beta\text{-}SbW_9]$ (193 mg, 0.034 mmol) and $CdCl_2$ (39.96 mg, 0.218 mmol) were dissolved in sodium acetate buffer (15 ml, pH 5.5, NaOAc/AcOH 0.5 M). The reaction mixture was heated to 80 °C for 2 h. A solution of tetra-methylammonium chloride (TMACl) (3 ml, [0.4 M]) was added dropwise to the hot reaction mixture, which was filtered after cooling to room temperature. Evaporation of the resulting orange solution at 18°C gave dark yellow block shaped crystals of $(TMA)_2Na_6H_8[(Mn(H_2O)_3)_2(CdCl_{0.8}O_{1.2})_2(B\text{-}\beta\text{-}SbW_9O_{33})_2]$ $[\beta\text{-}Cd_2Mn_2SbW_9]$ (**3**) ((yield 51 % based on W) after one week. Calcd (found): Mn, 1.3 (1.9); Cd, 3.7 (3.3); Sb, 3.9 (3.5); W, 53.8 (51.4).

3. IR-spectra

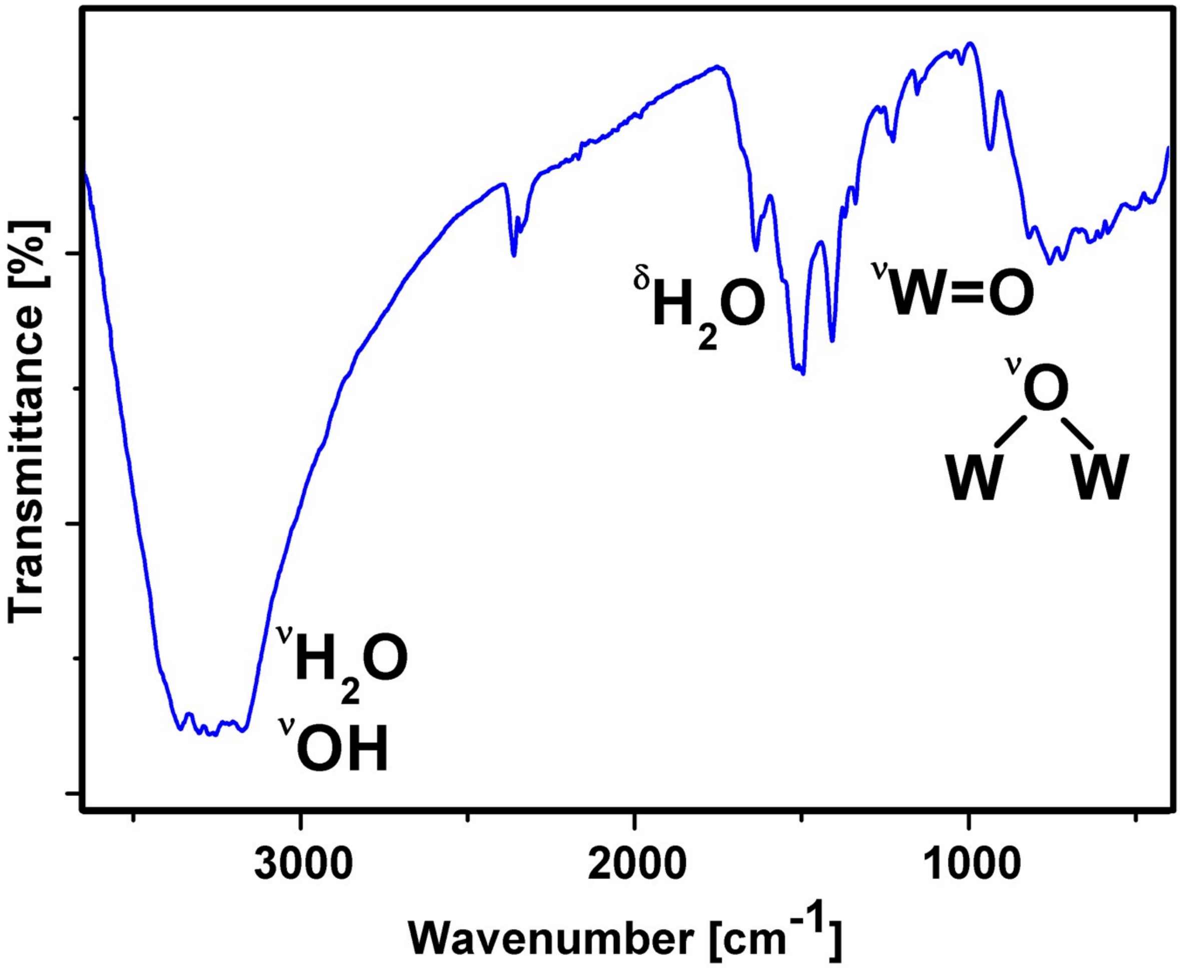

Figure S1: IR-spectrum of $(C_{12}N_4H_{11})_4K_4[(Mn(H_2O)_3)_2(Mn_{0.5}W_{0.5}O_{0.5})_2(B\text{-}\beta\text{-}SbW_9O_{33})_2]$ [Mn-β-SbW$_9$] (ATR-IR, cm^{-1}): 3364.4 (s), 3263.6 (s), 2359.4 (w), 1637.8 (w), 1508.1 (m), 1401.6 (m), 1234.6 (m), 1151.6 (w), 936.4 (w), 748.5 (m).

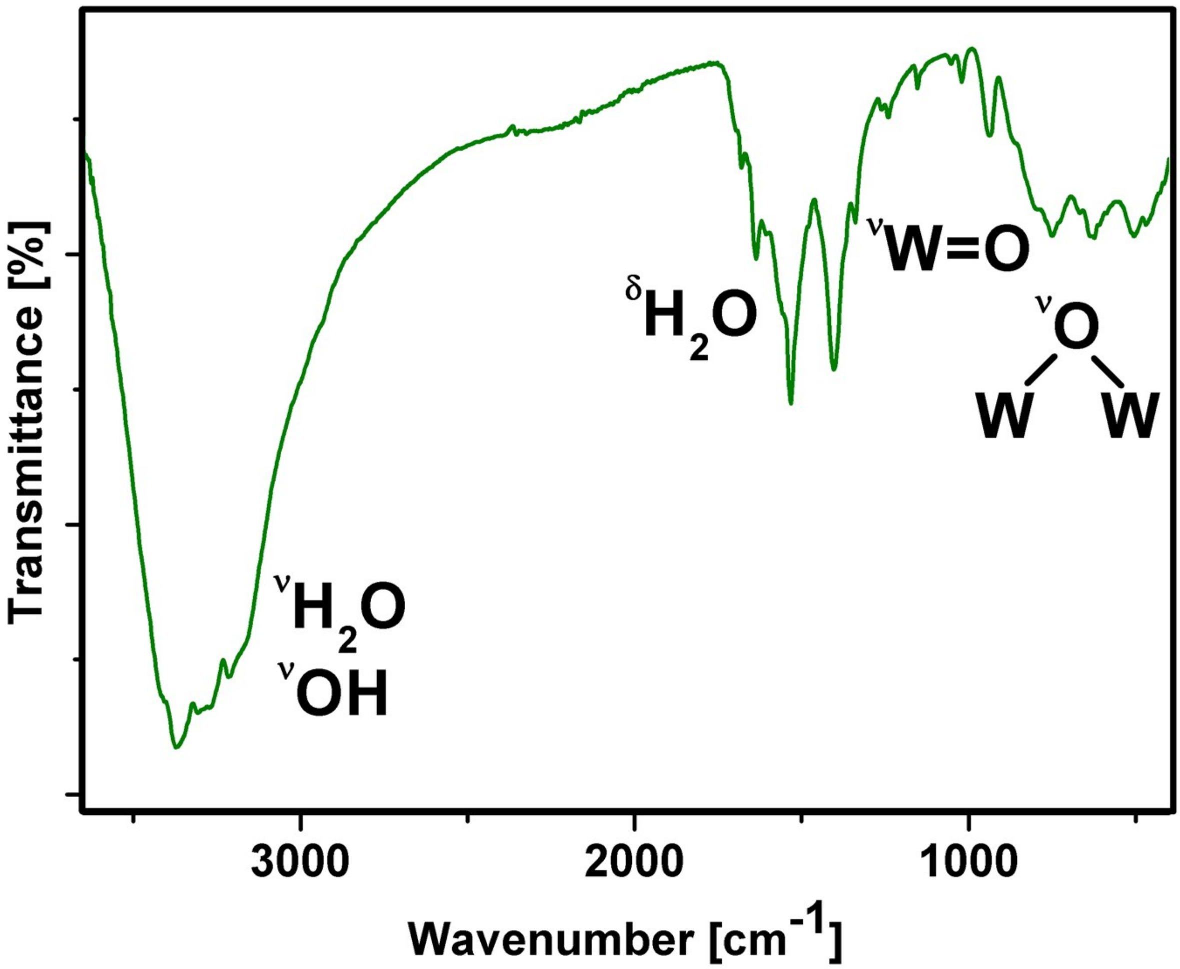

Figure S2: IR-spectrum of $(C_{12}N_4H_{11})_4Na_5[(Zn_{0.8}W_{0.2}(H_2O)_3)_2(Zn_{0.2}W_{0.8}O_2)_2(B\text{-}\beta\text{-}SbW_9O_{33})_2]$ $[(Zn/W)_2\text{-}\beta\text{-}SbW_9]$ (**2**) (ATR-IR, cm^{-1}): 3364.2 (s), 3206.7 (s), 1634.9 (m), 1530.3 (m), 1394.5 (m), 1326.9 (w), 1243.9 (w), 1153.9 (w), 1019.0 (w), 935.9 (w), 748.3 (w), 628.0 (w), 507.8 (w).

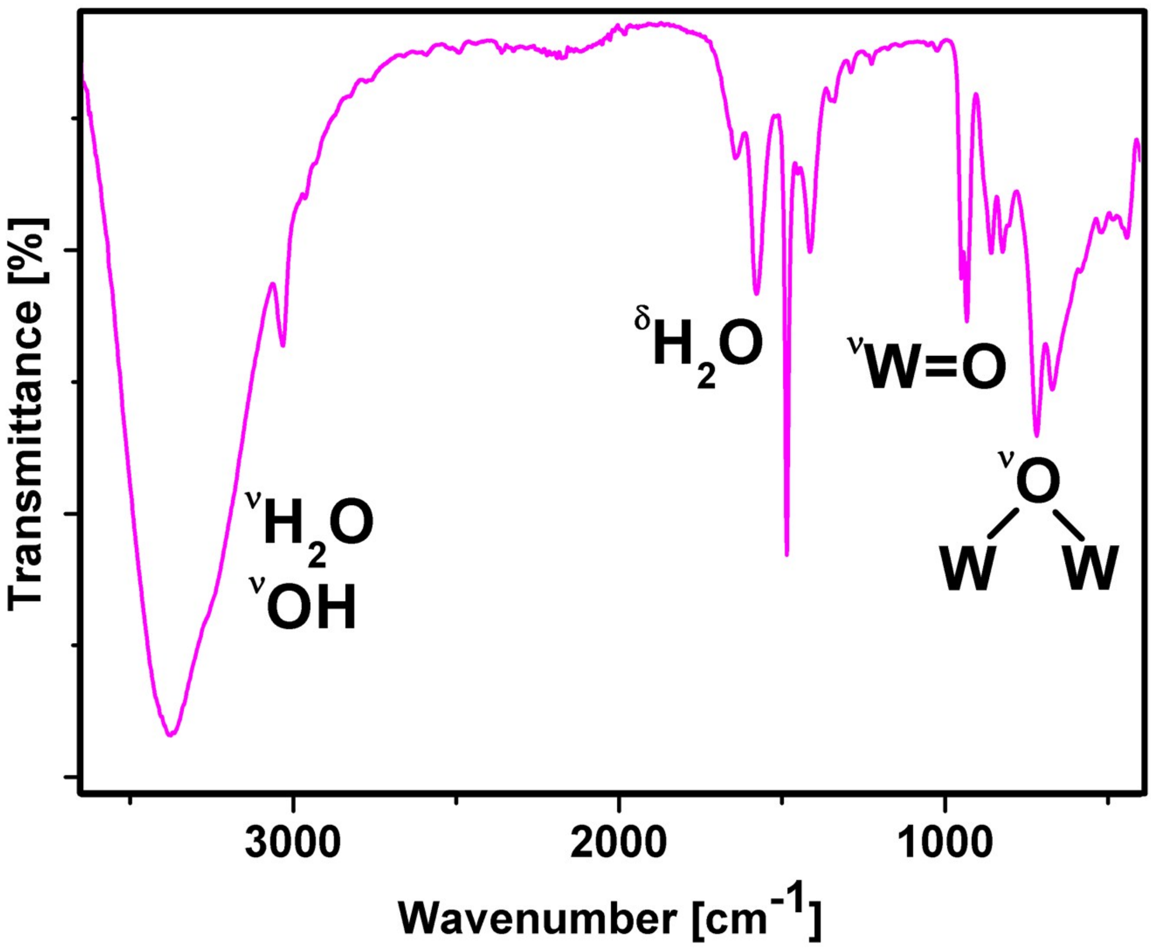

Figure S3: IR-spectrum of (TMA)($C_{12}N_4H_{11}$)$_5$[(Zn(H_2O))$_6$(*B*-α-SbW_9O_{33})$_2$] (TMA)($C_{12}N_4H_{11}$)$_5$[Zn_6-α-SbW_9] (**1**) (ATR-IR, cm^{-1}): 3364.8 (s), 3028 (m), 1637.4 (w), 1571.9 (m), 1485.1 (s), 1413.7 (m), 931.6 (m), 856.3 (w), 819.7 (w), 721.3 (m), 673.1 (m), 437.8 (w), 387.7 (w). TMA = tetra-methylammonium

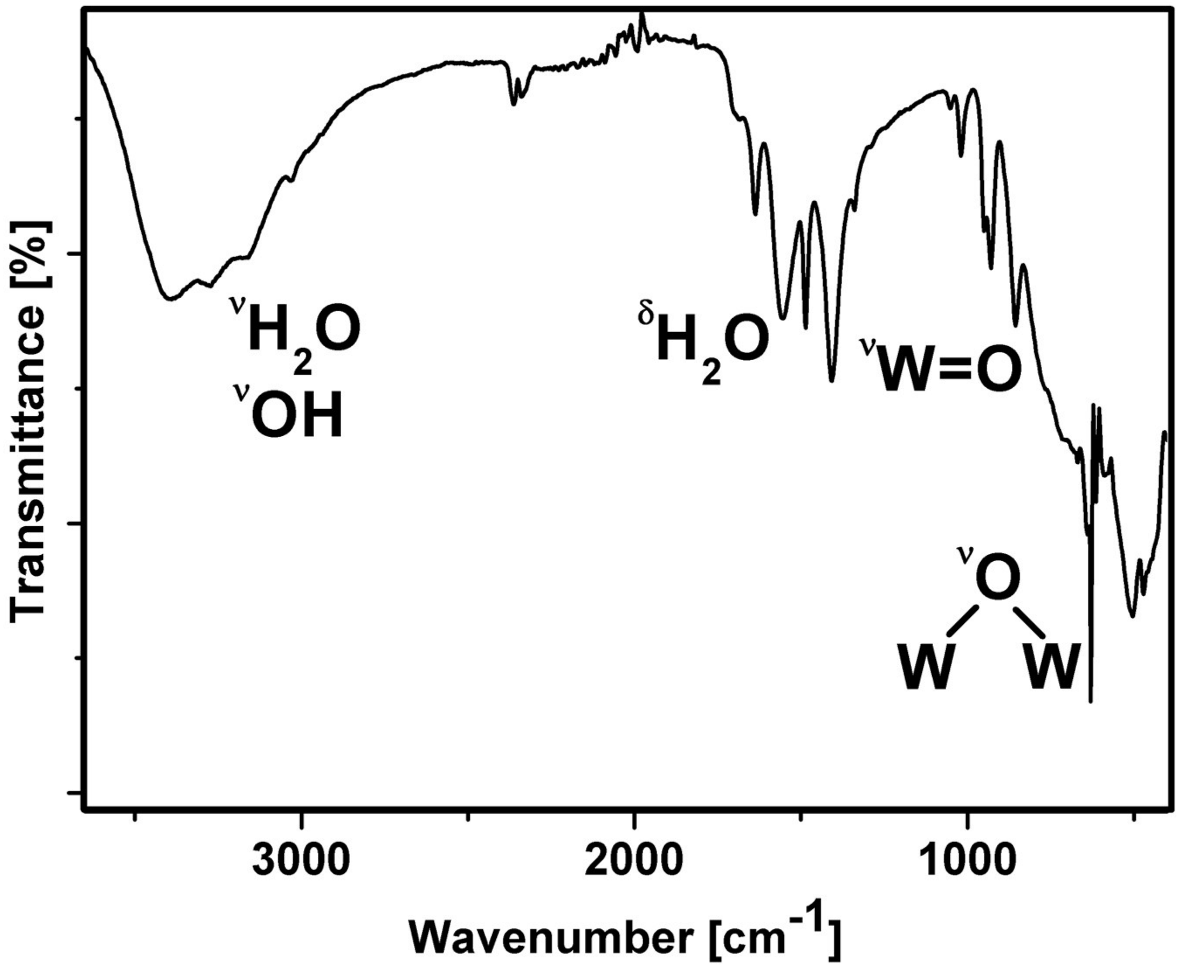

Figure S4: IR-spectrum of $(TMA)_6[(Zn(H_2O))_6(B\text{-}\alpha\text{-}SbW_9O_{33})_2]$ $(TMA)_6[Zn_6\text{-}\alpha\text{-}SbW_9]$ (**1**) (ATR-IR, cm^{-1}): 3365.5 (w), 3031.78 (w), 1579.53 (m), 1486.1 (m), 1413.9 (m), 1342.31 (w), 931.9 (s), 856.5 (m), 821.59 (m), 713.6 (m), 673.1 (m), 437.9 (w), 388.4 (w).TMA = tetra-methylammonium

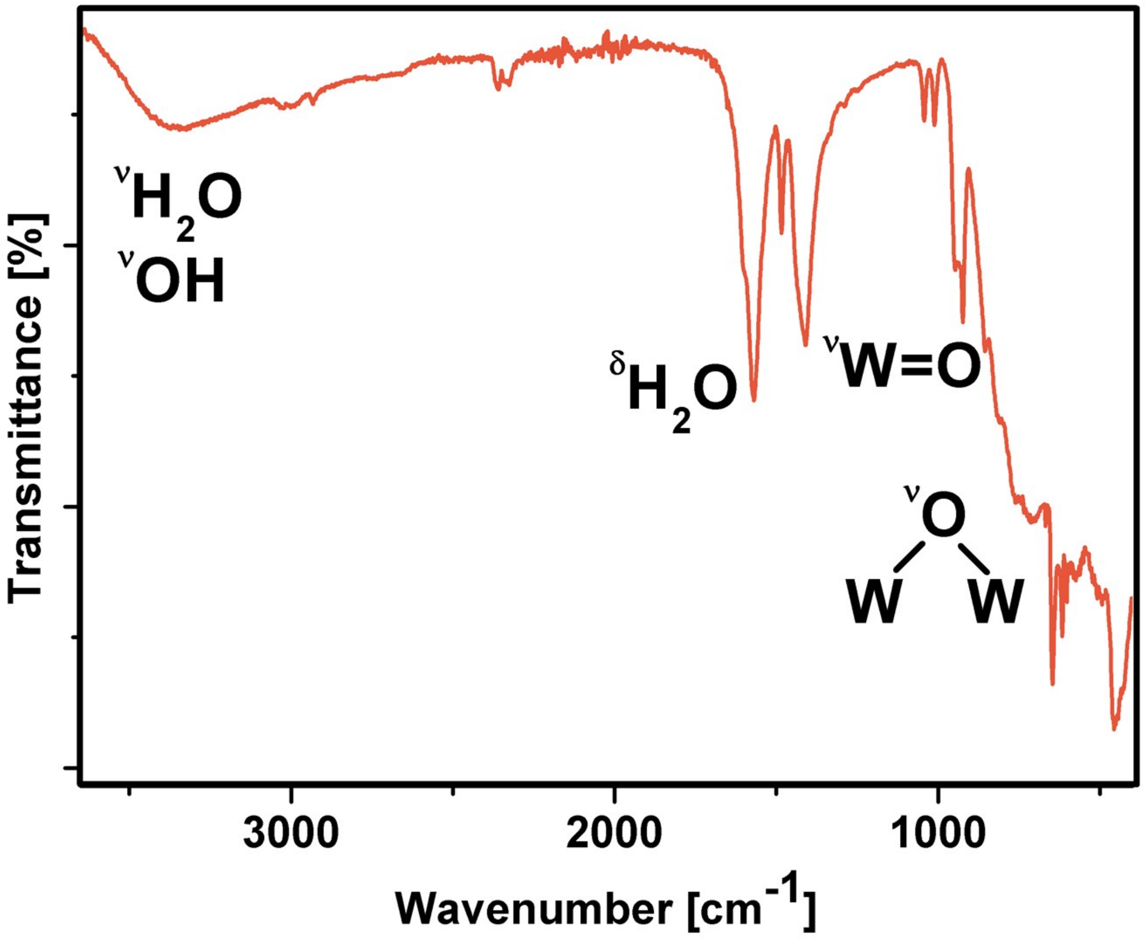

Figure S5: IR-spectrum of [Cd_2Mn_2-β-SbW_9] (**3**) (ATR-IR, cm^{-1}): 3365.8 (s), 3026 (m), 1637.4 (w), 1569.9 (m), 1483.1 (s), 1412.5 (m), 930.4 (m), 855.0 (w), 820.0 (w), 720.9 (m), 672.0 (m), 435.9 (w), 386.9 (w).

4. Thermogravimetric Analysis

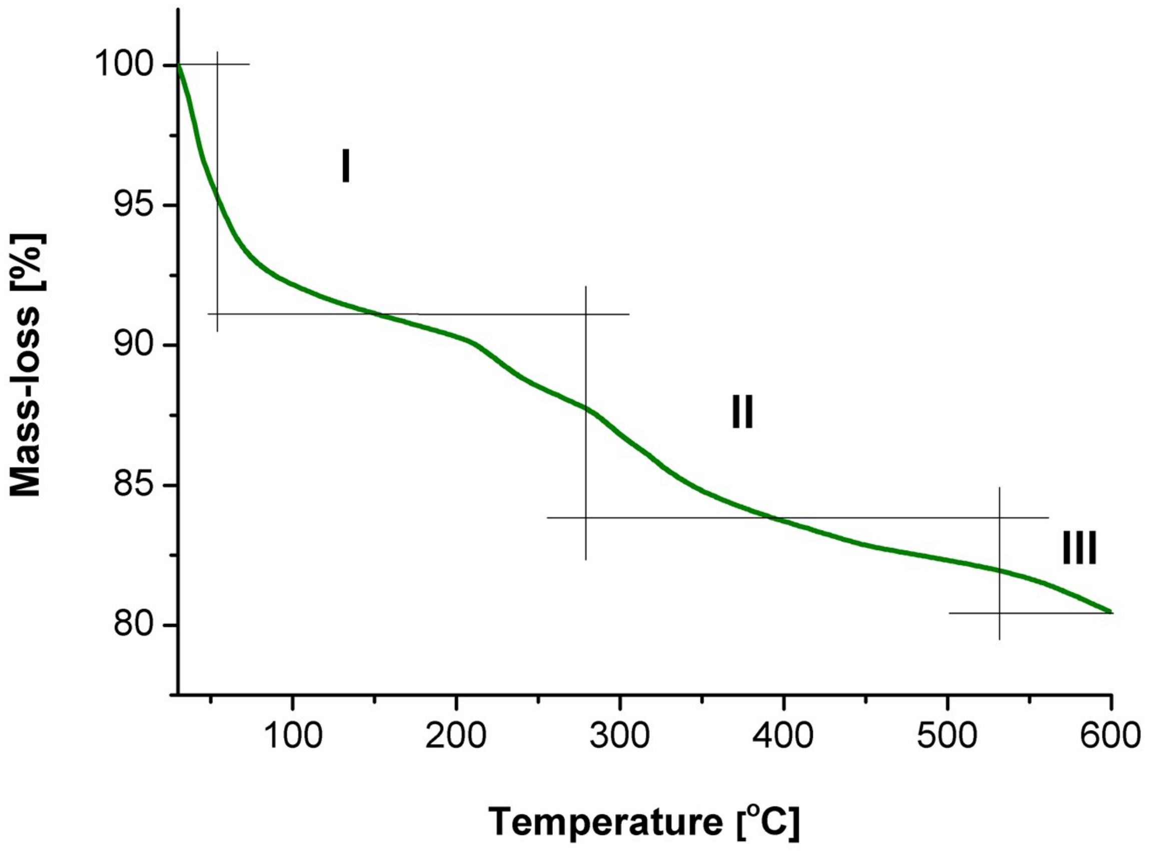

Figure S6: Thermogravimetric curve of $(C_{12}N_4H_{11})_4Na_5[(Zn_{0.8}W_{0.2}(H_2O)_3)_2(Zn_{0.2}W_{0.8}O_2)_2(B\text{-}\beta\text{-}SbW_9O_{33})_2]\bullet 13\ H_2O$ (**2**); DAP = 2, 3 - diaminophenazine

Table S2: TGA results for compound $(C_{12}N_4H_{11})_4Na_5[(Zn_{0.8}W_{0.2}(H_2O)_3)_2(Zn_{0.2}W_{0.8}O_2)_2(B\text{-}\beta\text{-}SbW_9O_{33})_2]\bullet 13\ H_2O$ (**2**); DAP = 2, 3 - diaminophenazine

Step	Temperatur/°C	Mass-loss/%	Number of molecules corresponding to mass-loss
I	**30-157**	**8.97**	**12.74 H_2O+1.71 DAP**
II	**157-402**	**7.32**	**2.28 DAP**
III	**402-600**	**1.5**	**5.5 H_2O**

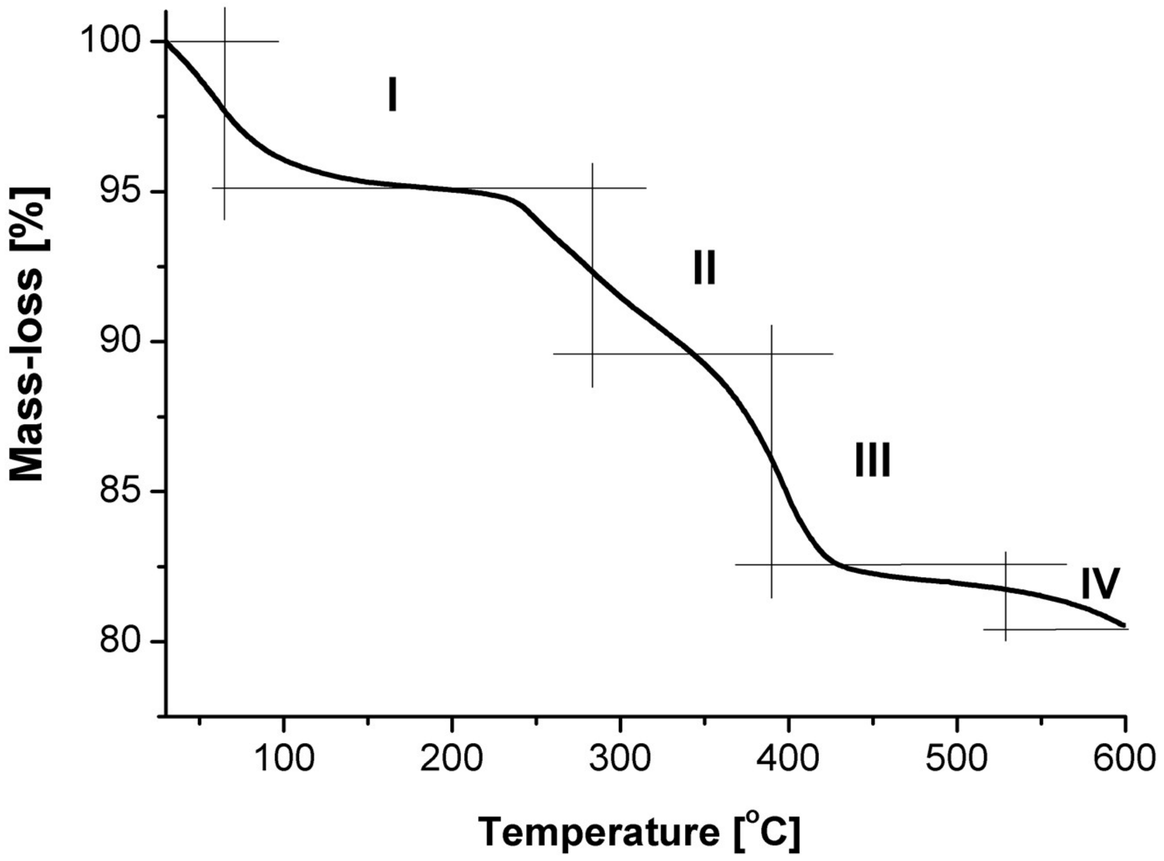

Figure S7: Thermogravimetric curve of $(TMA)_6[(Zn(H_2O))_6(B\text{-}\alpha\text{-}SbW_9O_{33})_2]\cdot 37.5\ H_2O$ (**1**); TMA = tetramethylammonium

Table S3: TGA results for compound $(TMA)_6[(Zn(H_2O))_6(B\text{-}\alpha\text{-}SbW_9O_{33})_2]\cdot 37.5\ H_2O$ (**1**); TMA = tetramethylammonium

Step	Temperatur/°C	Mass-loss/%	Number of molecules corresponding to mass-loss
I	**30-184**	**4.81**	**16.87 H_2O**
II	**184-342**	**5.59**	**20.7 H_2O**
III	**342-428**	**7.04**	**6.0 TMA**
IV	**428-600**	**2.06**	**7.2 H_2O**

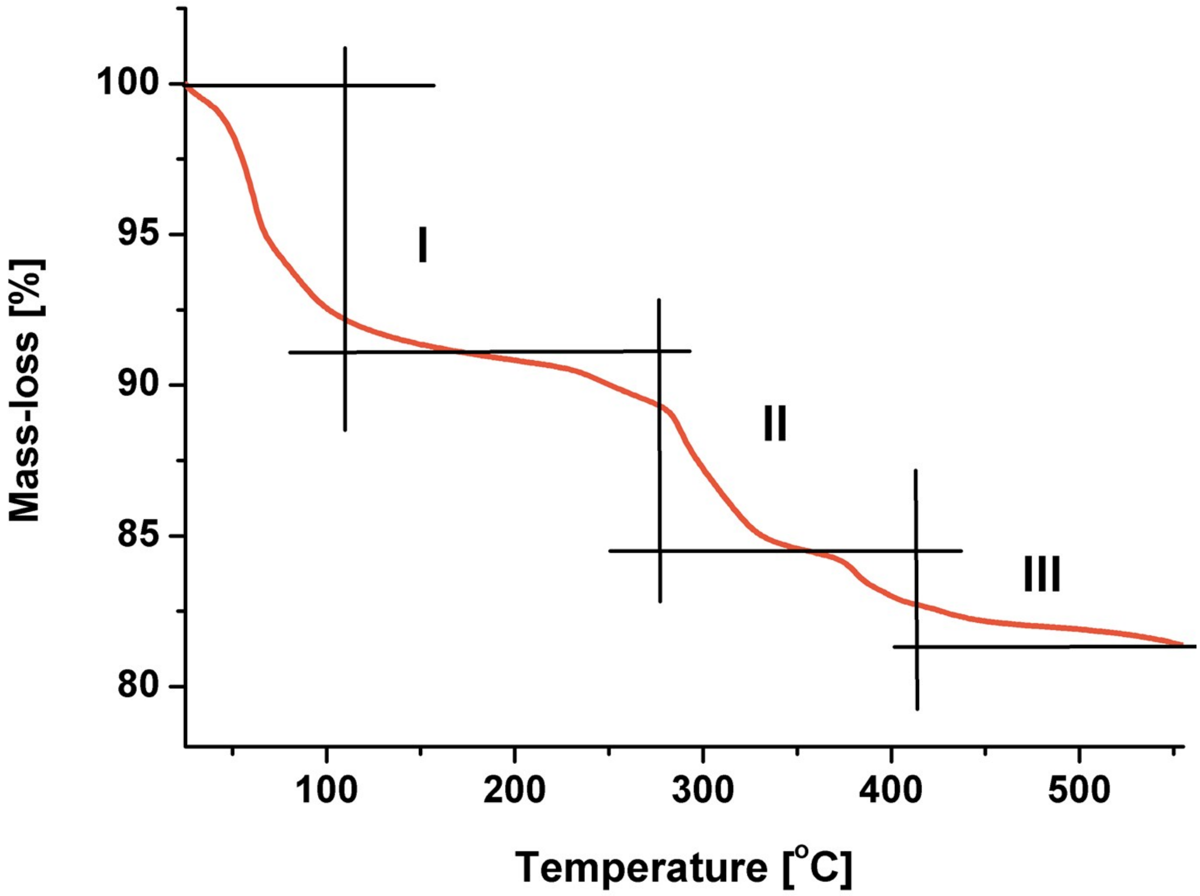

Figure S8: Thermogravimetric curve of $(TMA)_2Na_6H_8[(Mn(H_2O)_3)_2(CdCl_{0.8}O_{1.2})_2$(*B*-β-$SbW_9O_{33})_2]$•51.5 H_2O (**3**); TMA = tetramethylammonium

Table S4: TGA results for compound $(TMA)_2Na_6H_8[(Mn(H_2O)_3)_2(CdCl_{0.8}O_{1.2})_2$(*B*-β-$SbW_9O_{33})_2]$•51.5 H_2O (**3**); TMA = tetramethylammonium

Step	Temperatur/°C	Mass-loss/%	Number of molecules corresponding to mass-loss
I	**25-175**	**8.94**	**31.6 H_2O**
II	**175-350**	**6.48**	**14.73 H_2O + 2 TMA**
III	**350-550**	**3.14**	**5.9 H_2O + 4 Na**

5. Single-Crystal X-ray Diffraction

The X-ray intensity data were measured on Bruker X8 APEX2 equipped with multilayer monochromator and on Nonius Kappa CCD diffractometer equipped with a graphite monochromator, Mo K/α INCOATEC micro focus sealed tube and Kryoflex cooling device. The structures were solved by direct methods and refined by full-matrix least-squares techniques. Non-hydrogen atoms were refined with anisotropic displacement parameters. Hydrogen atoms were inserted at calculated positions and refined with a riding model. The following software was used: Bruker SAINT software package[4] using a narrow-frame

algorithm for frame integration, OLEX2[5] for structure solution, refinement, molecular diagrams and graphical user-interface, Shelxle[6] for refinement and graphical user-interface SHELXS-2013[7] for structure solution, SHELXL-2013[8] for refinement. Experimental data and CCDC-Codes can be found in Table S1. Crystal data, data collection parameters, and structure refinement details are given in Tables S2-S9.

Table S5: Experimental parameter and CCDC-Code.

Sample	Machine	Source	Temp.	Detector Distance	Time/ Frame	#Frames	Frame width	CCDC
			[K]	[mm]	[s]		[°]	
**[Mn-β-SbW_9]**	Nonius Kappa	Mo	200	35	85	404	2	1569737
**[$(Zn/W)_2$-β-SbW_9] (2)**	Bruker X8	Mo	200	40	80	623	2	1569739
**(TMA)$(C_{12}N_4H_{11})_5Zn_6$-α-SbW_9] (1)**	Bruker X8	Mo	100	35	60	2131	0.4	1569738
**$(TMA)_6[Zn_6$-α-SbW_9] (1)**	Bruker X8	Mo	100	35	5	5947	0.5	1576366
**[Cd2Mn2-β-SbW9] (3)**	Bruker X8	Mo	101.85	34	10	1215	0.5	1849600

Table S6: Sample and crystal data of **$(C_{12}N_4H_{11})_4K_4[(Mn(H_2O)_3)_2(Mn_{0.5}W_{0.5}O_{0.5})_2$(*B*-β-$SbW_9O_{33})_2$] [Mn-β-$SbW_9$]**

**Chemical formula**	$C_{48}H_{67.2}K_4Mn_3N_{16}O_{80.1}Sb_2W_{19}$	**Crystal system**	triclinic	
**Formula weight [g/mol]**	6207.84	**Space group**	*P-1*	
**Temperature [K]**	200	**Z**	1	
**Measurement method**	\w scans	**Volume [Å³]**	3477.6(14)	
**Radiation (Wavelength [Å])**	MoKα (λ = 0.71073)	**Unit cell dimensions [Å] and [°]**	12.116(2)	108.55(3)
**Crystal size / [mm³]**	0.05 × 0.05 × 0.05		14.374(3)	91.90(3)
**Crystal habit**	clear dark red block		21.414(4)	99.07(3)
**Density (calculated) / [g/cm³]**	2964	**Absorption coefficient / [mm⁻¹]**	16489	
**Abs. correction Tmin**	0.5616	**Abs. correction Tmax**	0.7536	
**Abs. correction type**	multi-scan	**F(000) [e⁻]**	2767.0	

Table S7: Data collection and structure refinement of **$(C_{12}N_4H_{11})_4K_4[(Mn(H_2O)_3)_2(Mn_{0.5}W_{0.5}O_{0.5})_2($*B*$\text{-}\beta\text{-}SbW_9O_{33})_2]$ [Mn-β-SbW_9]**

**Index ranges**	-17 ≤ h ≤ 16, -20 ≤ k ≤ 20, -29 ≤ l ≤ 29	**Theta range for data collection [°]**	4.15 to 60.128	
**Reflections number**	37980	**Data / restraints / parameters**	20123/25/800	
**Refinement method**	Least squares	**Final R indices**	all data	R1 = 0.1026, wR2 = 0.1451
**Function minimized**	Σ w(F_o^2 - F_c^2)^2		I>2σ(I)	R1 = 0.0553, wR2 = 0.1289
**Goodness-of-fit on F^2**	1017	**Weighting scheme**	w=1/[$\sigma^2(F_o^2)+(0.0702P)^2$]	
**Largest diff. peak and hole [e Å^{-3}]**	3.18/-2.69		where P=(F_o^2+2F_c^2)/3	

Table S8: Sample and crystal data of **$(C_{12}N_4H_{11})_4Na_5[(Zn_{0.8}W_{0.2}(H_2O)_3)_2(Zn_{0.2}W_{0.8}O_2)_2($*B*$\text{-}\beta\text{-}SbW_9O_{33})_2]$ [(Zn/W)$_2$-β-SbW_9]** (**2**)

**Chemical formula**	$C_{48}H_{53}N_{16}Na_5O_{83}Sb_2W_{19.89}Zn_{2.11}$	**Crystal system**	triclinic	
**Formula weight [g/mol]**	6335.22	**Space group**	*P-1*	
**Temperature [K]**	200.0	**Z**	1	
**Measurement method**	\w scans	**Volume [Å^3]**	3303.7(3)	
**Radiation (Wavelength [Å])**	MoKα (λ = 0.71073)	**Unit cell dimensions [Å] and [°]**	12.6796(7)	92.665(3)
**Crystal size / [mm^3]**	0.125 × 0.05 × 0.01		14.1057(7)	93.300(3)
**Crystal habit**	clear red plate		20.5025(11)	115.147(3)
**Density (calculated) / [g/cm^3]**	3184	**Absorption coefficient / [mm^{-1}]**	18120	
**Abs. correction Tmin**	0.4500	**Abs. correction Tmax**	0.7461	
**Abs. correction type**	multi-scan	**F(000) [e$^-$]**	2809.0	

Table S9: Data collection and structure refinement of **$(C_{12}N_4H_{11})_4Na_5[(Zn_{0.8}W_{0.2}(H_2O)_3)_2(Zn_{0.2}W_{0.8}O_2)_2(\textit{B}\text{-}\beta\text{-}SbW_9O_{33})_2]$ $[(Zn/W)_2\text{-}\beta\text{-}SbW_9]$** (**2**)

**Index ranges**	-15 ≤ h ≤ 15, -16 ≤ k ≤ 16, -24 ≤ l ≤ 24	**Theta range for data collection [°]**	4.23 to 50.696	
**Reflections number**	65919	**Data / restraints / parameters**	12045/37/798	
**Refinement method**	Least squares	**Final R indices**	all data	R1 = 0.0519, wR2 = 0.0956
**Function minimized**	Σ w(F_o^2 - F_c^2)^2		I>2σ(I)	R1 = 0.0403, wR2 = 0.0915
**Goodness-of-fit on F^2**	1018	**Weighting scheme**	w=1/[σ^2(F_o^2)+(0.0329P)^2+128.4466P]	
**Largest diff. peak and hole [e Å^{-3}]**	3.72/-1.82		where P=(F_o^2+2F_c^2)/3	

Table S10: Sample and crystal data of **$(TMA)(C_{12}N_4H_{11})_5[(Zn(H_2O))_6(\textit{B}\text{-}\alpha\text{-}SbW_9O_{33})_2]$ $(TMA)(C_{12}N_4H_{11})_5[Zn_6\text{-}\alpha\text{-}SbW_9]$** (**1**)

	$C_{68}H_{109}N_{22}O_{94}Sb_2W_{18}Zn_6$	**Crystal system**	triclinic	
**Formula weight [g/mol]**	6683.79	**Space group**	*P-1*	
**Temperature [K]**	100.0	**Z**	1	
**Measurement method**	\w scans	**Volume [Å^3]**	3467.5(4)	
**Radiation (Wavelength [Å])**	MoKα (λ = 0.71073)	**Unit cell dimensions [Å] and [°]**	12.4792(8)	94.425(4)
**Crystal size / [mm^3]**	0.14 × 0.07 × 0.03		17.4813(13)	109.140(3)
**Crystal habit**	clear red block		17.6938(11)	104.946(4)
**Density (calculated) / [g/cm^3]**	3201	**Absorption coefficient / [mm^{-1}]**	16367	
**Abs. correction Tmin**	0.4281	**Abs. correction Tmax**	0.7460	
**Abs. correction type**	multi-scan	**F(000) [e$^-$]**	3037.0	

Table S11: Data collection and structure refinement of **$(TMA)(C_{12}N_4H_{11})_5[(Zn(H_2O))_6(\textit{B}\text{-}\alpha\text{-}SbW_9O_{33})_2]$ $(TMA)(C_{12}N_4H_{11})_5[Zn_6\text{-}\alpha\text{-}SbW_9]$** (**1**)

**Index ranges**	$-15 \leq h \leq 15$, $-21 \leq k \leq 21$, $-21 \leq l \leq 21$	**Theta range for data collection [°]**	4.688 to 50.7	
**Reflections number**	56012	**Data / restraints / parameters**	12509/108/973	
**Refinement method**	Least squares	**Final R indices**	all data	R1 = 0.0683, wR2 = 0.1124
**Function minimized**	$\Sigma\ w(F_o^2 - F_c^2)^2$		$I>2\sigma(I)$	R1 = 0.0449, wR2 = 0.1007
**Goodness-of-fit on F^2**	1027	**Weighting scheme**	$w=1/[\sigma^2(F_o^2)+(0.0432P)^2+53.8347P]$	
**Largest diff. peak and hole [e Å^{-3}]**	2.10/-1.89		where $P=(F_o^2+2F_c^2)/3$	

Table S12: Sample and crystal data of **$(TMA)_6[(Zn(H_2O))_6(\mathit{B}\text{-}\alpha\text{-}SbW_9O_{33})_2]$ $(TMA)_6[Zn_6\text{-}\alpha\text{-}SbW_9]$** (**1**)

**Chemical formula**	$C_{16}H_{48}N_4O_{72}Sb_2W_{18}Zn_6$	**Crystal system**	monoclinic	
**Formula weight [g/mol]**	5393.60	**Space group**	*C2/m*	
**Temperature [K]**	100	**Z**	2	
**Measurement method**	f and w scans	**Volume [Å^3]**	6060.2(7)	
**Radiation (Wavelength [Å])**	MoKα (λ = 0.71073)	**Unit cell dimensions [Å] and [°]**	14.5029(12)	90.0
**Crystal size / [mm^3]**	0.3 × 0.28 × 0.24		23.1699(12)	95.440(4)
**Crystal habit**	clear colorless block		18.1162(11)	90.0
**Density (calculated) / [g/cm^3]**	2956	**Absorption coefficient / [mm^{-1}]**	18674	
**Abs. correction Tmin**	0.1335	**Abs. correction Tmax**	0.7460	
**Abs. correction type**	multi-scan	**F(000) [e$^-$]**	4724.0	

Table S13: Data collection and structure refinement of **$(TMA)_6[(Zn(H_2O))_6(\mathit{B}\text{-}\alpha\text{-}SbW_9O_{33})_2]$ $(TMA)_6[Zn_6\text{-}\alpha\text{-}SbW_9]$** (**1**)

**Index ranges**	$-19 \le h \le 19$, $-30 \le k \le 30$, $-23 \le l \le 23$	**Theta range for data collection [°]**	4.166 to 55.752	
**Reflections number**	151418	**Data / restraints / parameters**	7386/108/284	
**Refinement method**	Least squares	**Final R indices**	all data	R1 = 0.0783, wR2 = 0.2026
**Function minimized**	$\Sigma\ w(F_o^2 - F_c^2)^2$		I>2σ(I)	R1 = 0.0753, wR2 = 0.1982
**Goodness-of-fit on F^2**	1069	**Weighting scheme**	$w=1/[\sigma^2(F_o^2)+(0.1346P)^2+178.2807P]$	
**Largest diff. peak and hole [e Å^{-3}]**	3.34/-6.82		where $P=(F_o^2+2F_c^2)/3$	

Table S14: Sample and crystal data of **$(TMA)_2Na_6H_8[(Mn(H_2O)_3)_2(CdCl_{0.8}O_{1.2})_2(\mathit{B}\text{-}\beta\text{-}SbW_9O_{33})_2]$ $[Cd_2Mn_2\text{-}\beta\text{-}SbW_9]$** (**3**)

**Chemical formula**	$C_8H_{32}Cd_2ClMn_2N_2Na_6O_{93.5}Sb_2W_{18}$	**Crystal system**	triclinic	
**Formula weight [g/mol]**	5713,22	**Space group**	*P-1*	
**Temperature [K]**	101,85	**Z**	1	
**Measurement method**	f and w scans	**Volume [Å^3]**	2471.2(3)	
**Radiation (Wavelength [Å])**	MoKα (λ = 0.71073)	**Unit cell dimensions [Å] and [°]**	12.3753(7)	70.120(2)
**Crystal size / [mm^3]**	0.451 × 0.136 × 0.042		13.6520(8)	83.879(2)
**Crystal habit**	clear orange block		17.0910(10)	65.614(2)
**Density (calculated) / [g/cm^3]**	3,839	**Absorption coefficient / [mm^{-1}]**	22,216	
**Abs. correction Tmin**	0,0003	**Abs. correction Tmax**	0,0082	
**Abs. correction type**	multi-scan	**F(000) [e$^-$]**	2505	

Table S15: Data collection and structure refinement of **$(TMA)_2Na_6H_8[(Mn(H_2O)_3)_2(CdCl_{0.8}O_{1.2})_2(\mathit{B}\text{-}\beta\text{-}SbW_9O_{33})_2]$ $[Cd_2Mn_2\text{-}\beta\text{-}SbW_9]$** (**3**)

**Index ranges**	$-14 \le h \le 14$, $-16 \le k \le 16$, $-20 \le l \le 20$	**Theta range for data collection [°]**	4.498 to 50.7
**Reflections number**	42040	**Data / restraints / parameters**	8983/75/675

**Refinement method**	Least squares	**Final R indices**	all data	R1 = 0.0713, wR2 = 0.1780
**Function minimized**	Σ w(F_o^2 - F_c^2)^2		I>2σ(I)	R1 = 0.0652, wR2 = 0.1693
**Goodness-of-fit on F^2**	1,109	**Weighting scheme**	w=1/[$\sigma^2(F_o^2)$+(0.1280P)^2+11.0744P]	
**Largest diff. peak and hole [e Å^{-3}]**	3.97/-1.83		where P=(F_o^2+2F_c^2)/3	

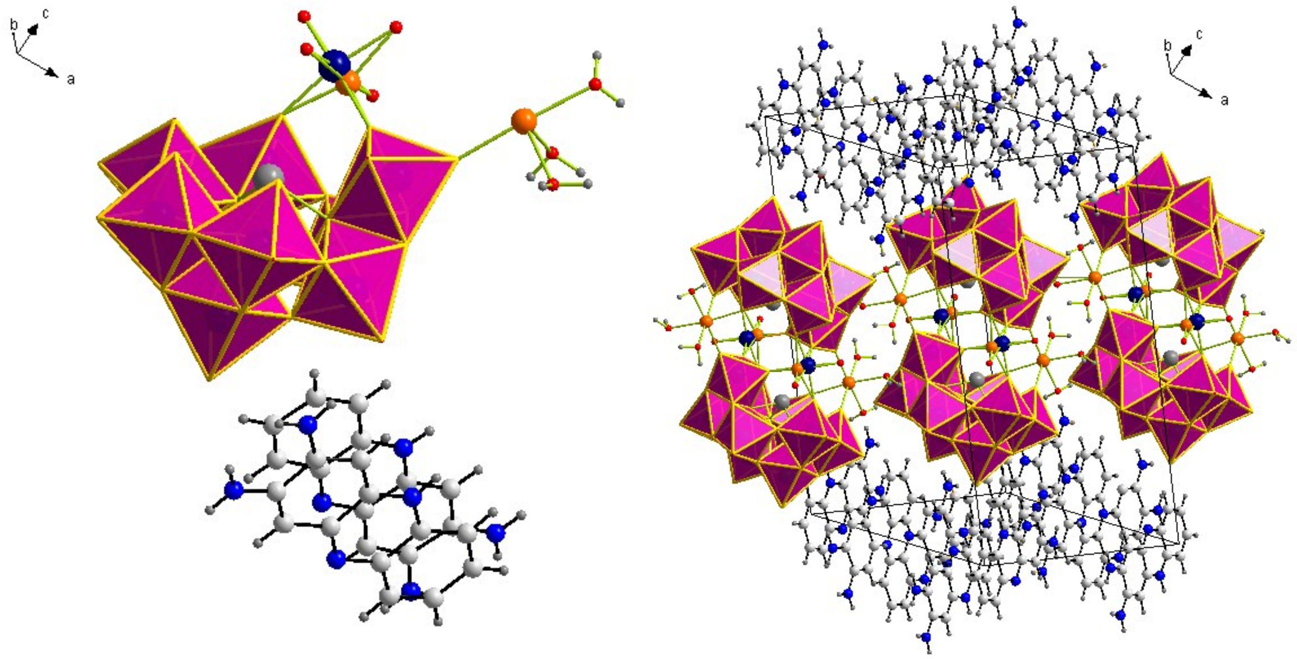

Figure S9: Asymmetric unit and packing of [Mn-β-SbW$_9$] (CCDC 1569737) along the z-axis. Color legend: WO$_6$, pink, octahedra; W, blue; O, red; C, grey; Mn, orange; N; dark blue; Sb, grey

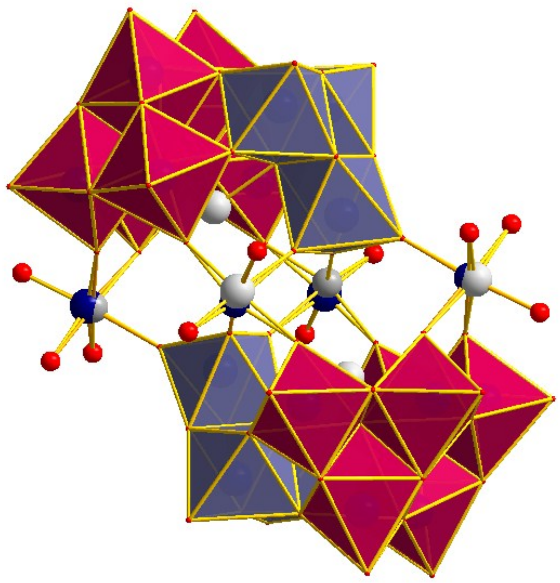

Figure S10: Polyhedral representation of $[(Zn/W)_2\text{-}\beta\text{-}SbW_9]$ (**2**) (CCDC 1569739) showing the four disordered Zn/W centers. Color legend: WO_6, pink; octahedral; WO_6 60° rotated, ice blue; W, dark blue; Zn, silver; Sb, white; O, red.

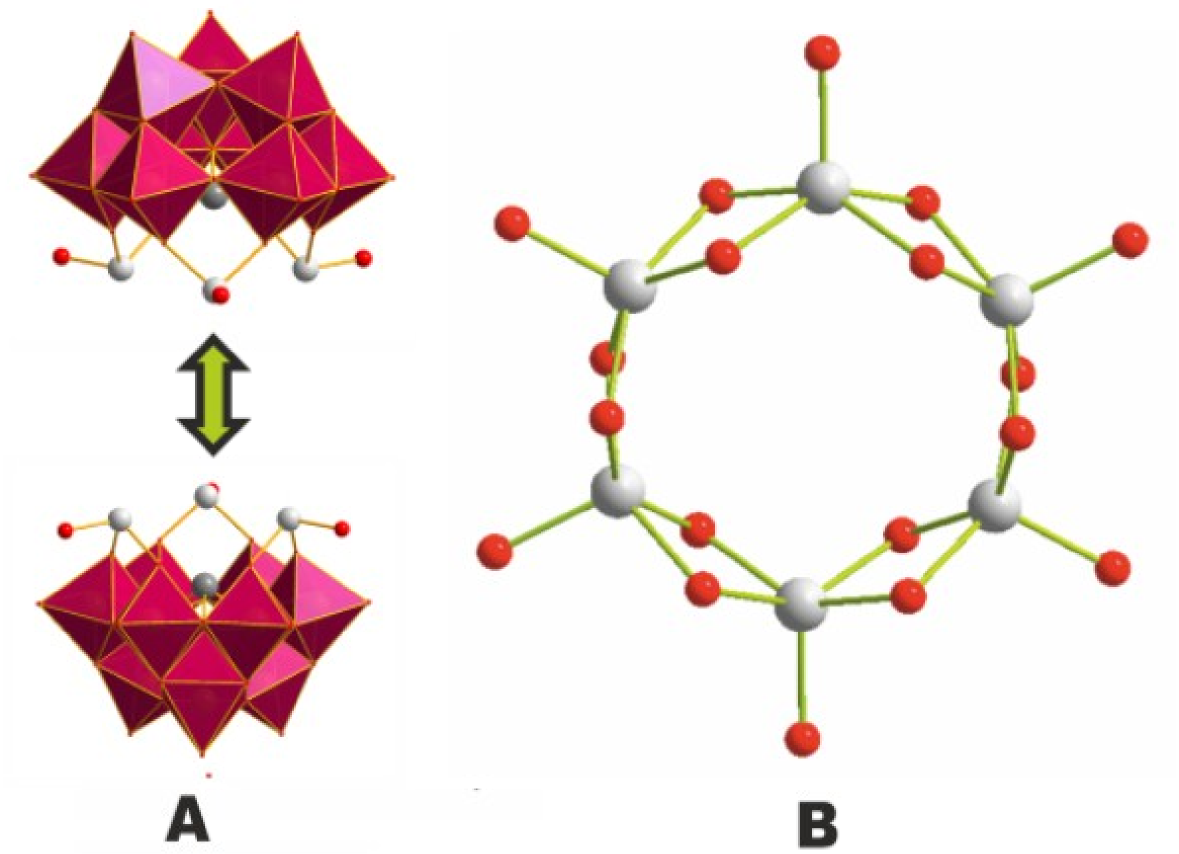

Figure S11: Polyhedral representation of $[Zn_6\text{-}\alpha\text{-}SbW_9]$ (**1**) (CCDC 1576366/CCDC 1569738) showing both $[(Zn(H_2O))_3(B\text{-}\beta\text{-}SbW_9O_{33})]$ building blocks related by an inversion center (A) and Zn_6-hexagon ring (B). Color legend: WO_6, pink, octahedra; O, red; Zn, silver; Sb, dark grey.

6 PXRD

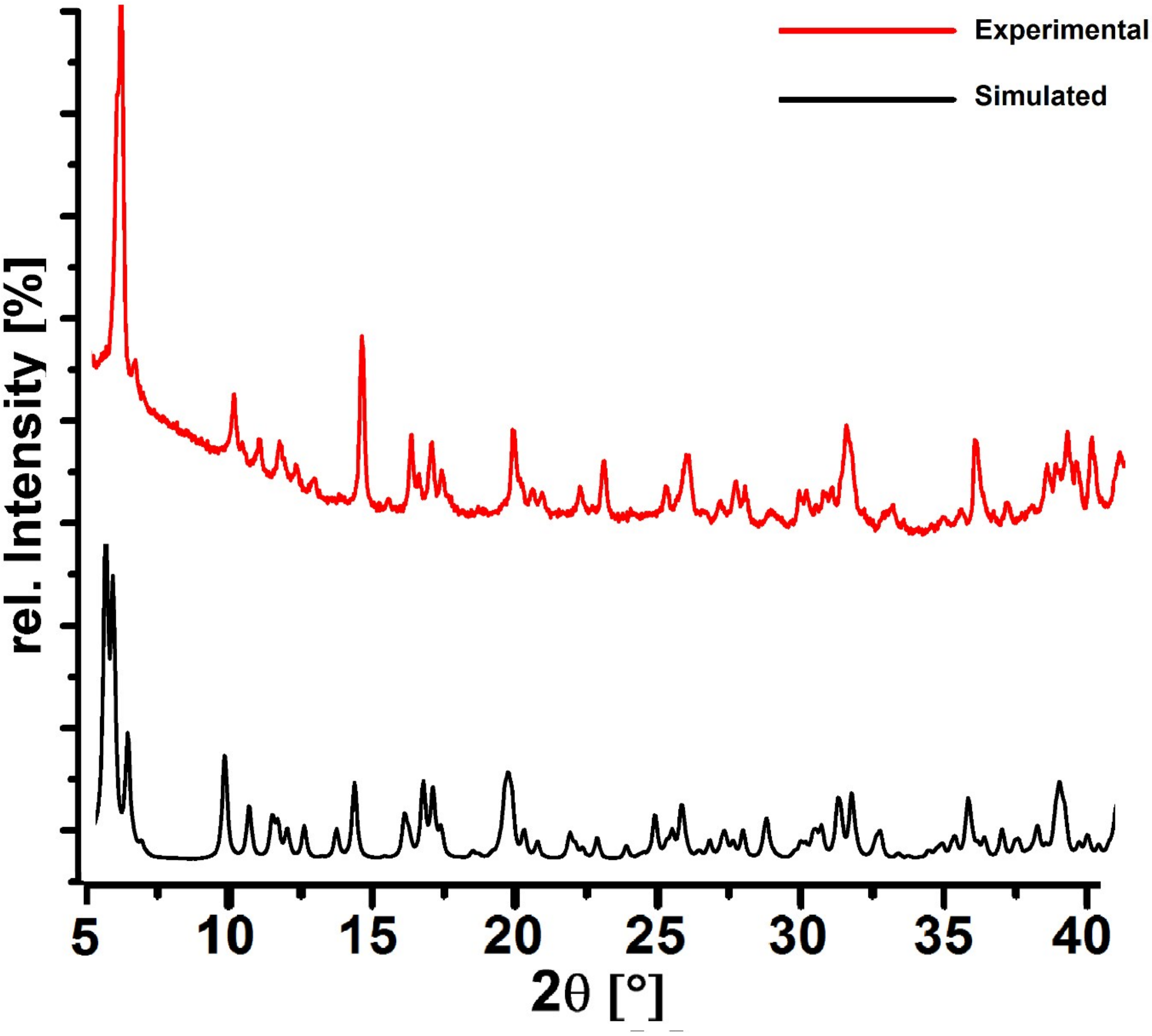

Figure S12: Comparison of the experimental and simulated PXRD patterns of [(Zn/W)$_2$-β-SbW$_9$] (**2**).

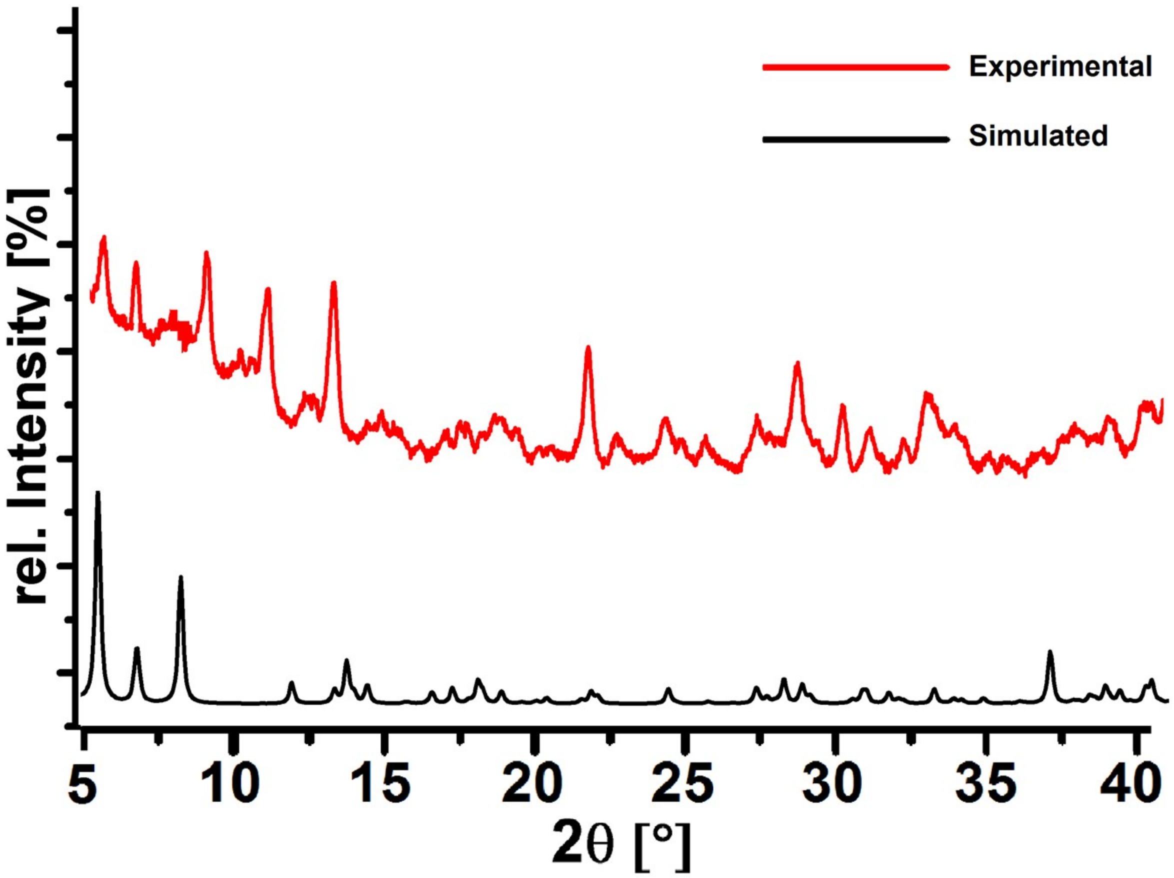

Figure S13: Comparison of the experimental and simulated PXRD patterns of $(TMA)_6[Zn_6$-α-$SbW_9]$ (**1**).

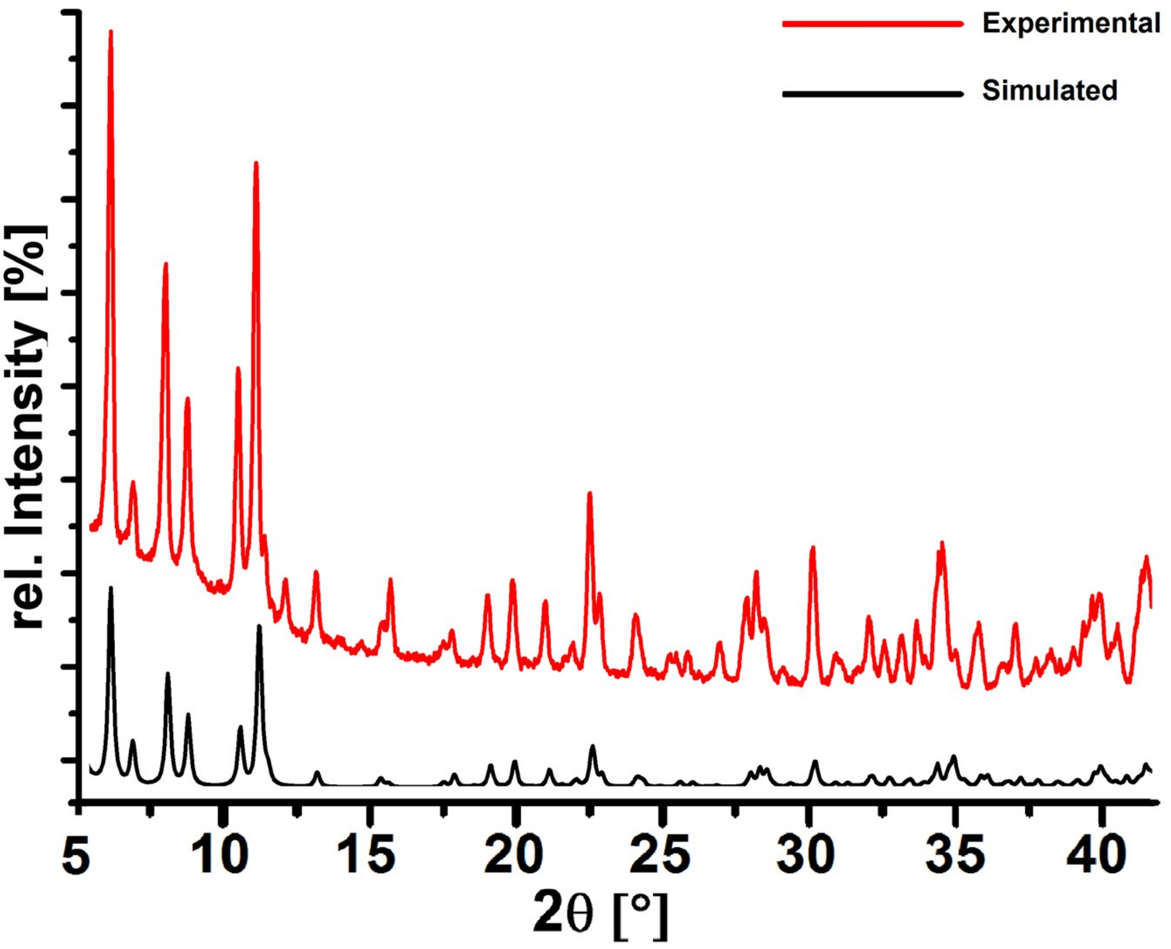

Figure S14: Comparison of the experimental and simulated PXRD patterns of [Cd_2Mn_2-β-SbW_9] (**3**).

7 UV-vis Spectroscopy

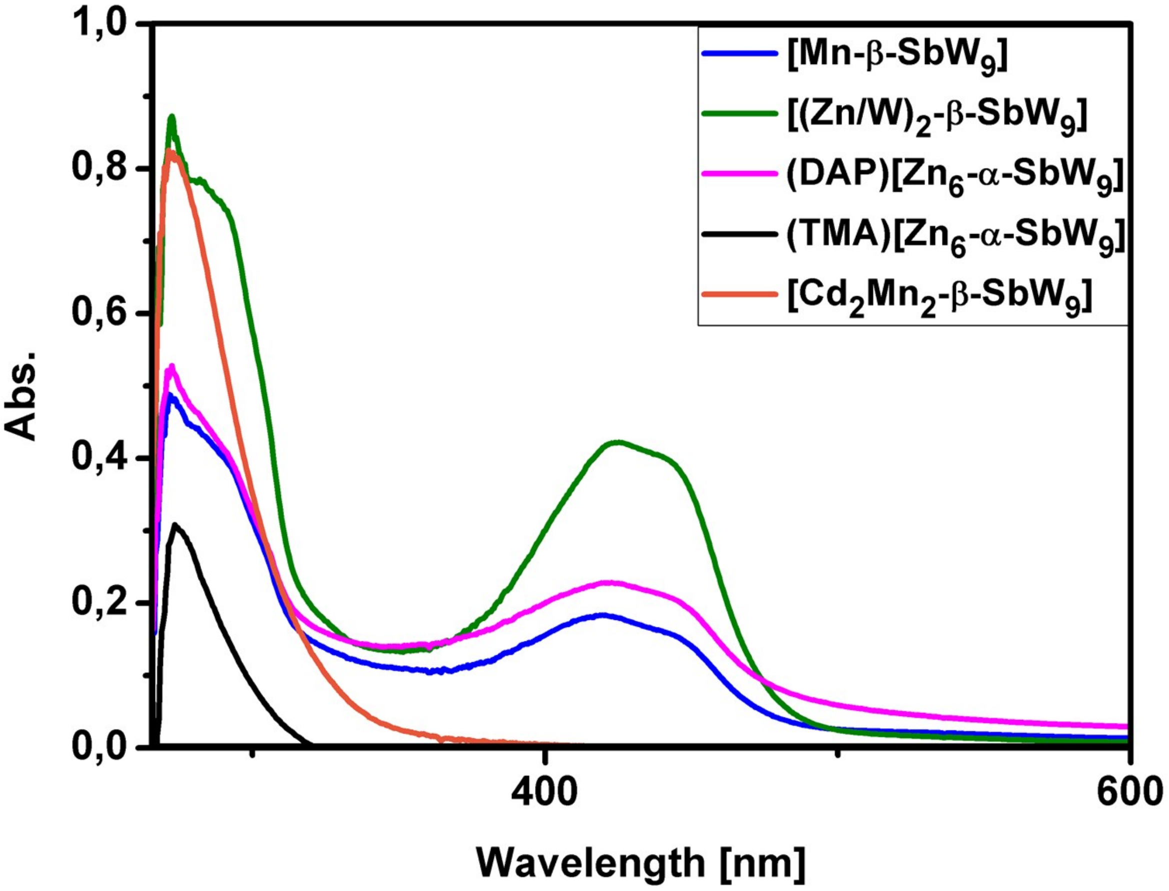

Figure S15: UV/Vis-spectrum of [Mn-β-SbW_9], [$(Zn/W)_2$-β-SbW_9] (**2**), (TMA)$(C_{12}N_4H_{11})_5$[Zn_6-α-SbW_9] (**1**), $(TMA)_6$[Zn_6-α-SbW_9] (**1**) and [Cd_2Mn_2-β-SbW_9] (**3**) showing typical O→W ligand−to−metal charge−transfer (272 nm) and aromatic transitions (425 nm)

Table S16: Concentrations of corresponding POM compound in sodium acetate buffer (pH 5.5, NaOAc/AcOH 10 mM). (Fig. S15).

POM compound	Concentration [mg/mL]
**[Mn-β-SbW_9]**	**0.30**
**[$(Zn/W)_2$-β-SbW_9]** (**2**)	**0.04**
**(TMA)$(C_{12}N_4H_{11})_5$[Zn_6-α-SbW_9]** (**1**)	**0.55**
**$(TMA)_6$[Zn_6-α-SbW_9]** (**1**)	**0.09**
**[Cd_2Mn_2-β-SbW_9]** (**3**)	**0.61**

8 ESI-Mass Spectrometry

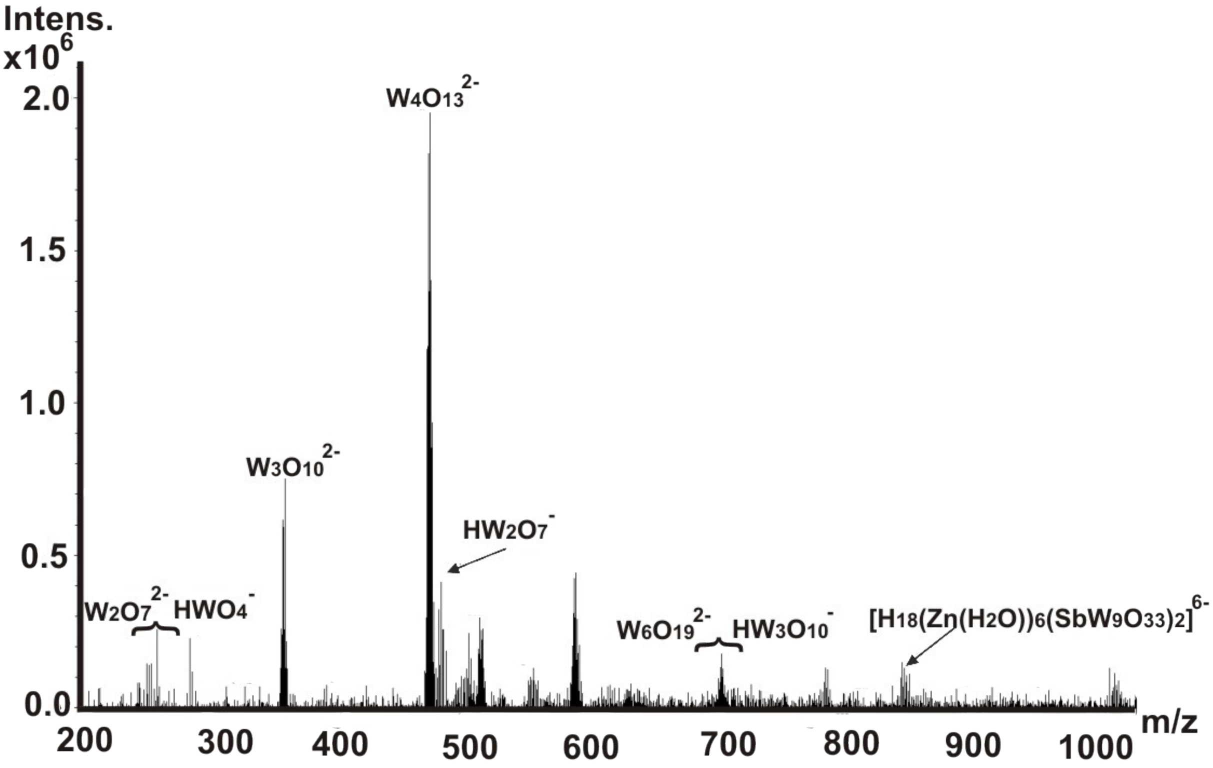

Figure S16: ESI-mass spectrum of (TMA)$_6$[Zn$_6$-α-SbW$_9$] (**1**) revealing W-containing decomposition products based on the polyanion.

Table S17: Species assigned to the peaks in the ESI-MS spectrum of (TMA)$_6$[(Zn(H$_2$O))$_6$(*B*-α-SbW$_9$O$_{33}$)$_2$]•37.5 H$_2$O (**1**) (Fig. S16).

m/z	m/z (calc.)	Peak Assignment
**239.74**	239.93	**$[W_2O_7]^{2-}$**
**248.8**	248.80	**$H[WO_4]^{-}$**
**355.8**	355.80	**$[W_3O_{10}]^{2-}$**
**471.75**	471.75	**$[W_4O_{13}]^{2-}$**
**480.74**	480.87	**$H[W_2O_7]^{2-}$**
**703.56**	703.80	**$[W_6O_{19}]^{2-}$**
**712.5**	712.50	**$H[W_3O_{10}]^{-}$**
**851.48**	851.54	**$[(Zn(H_2O))_6(SbW_9O_{33})_2]^{6-}$**

9 POM-Protein Interactions

SDS−PAGE: Human serum albumin (HSA) (5 µg) was dissolved with 1, 10, and 100 equivalents of corresponding POM in 25% (v/v) DMSO solution (Fig. S17 A-F) or 10 mM NaOAc buffer pH 5.5 (Fig. S17 G-N), respectively, and incubated at three different temperatures.

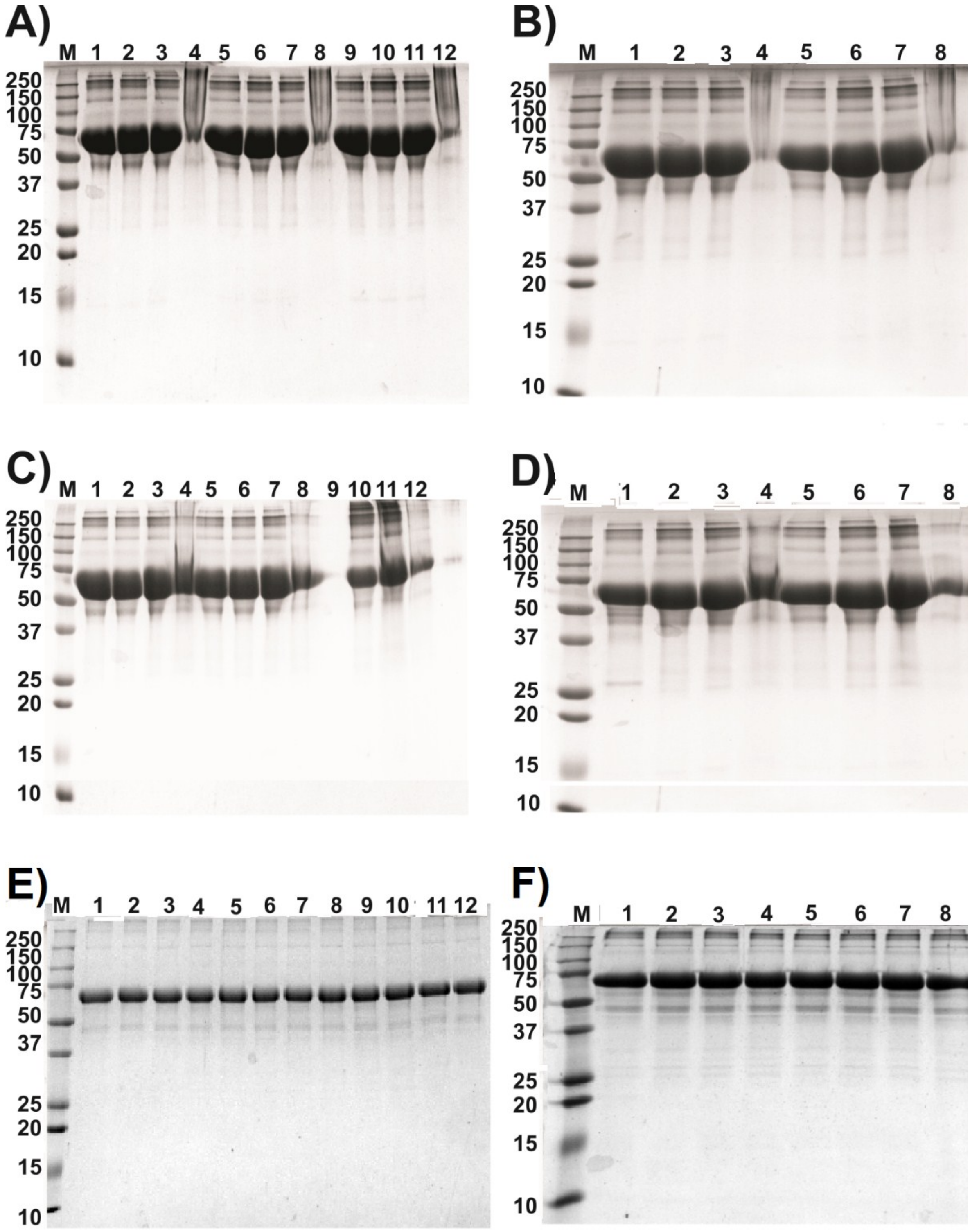

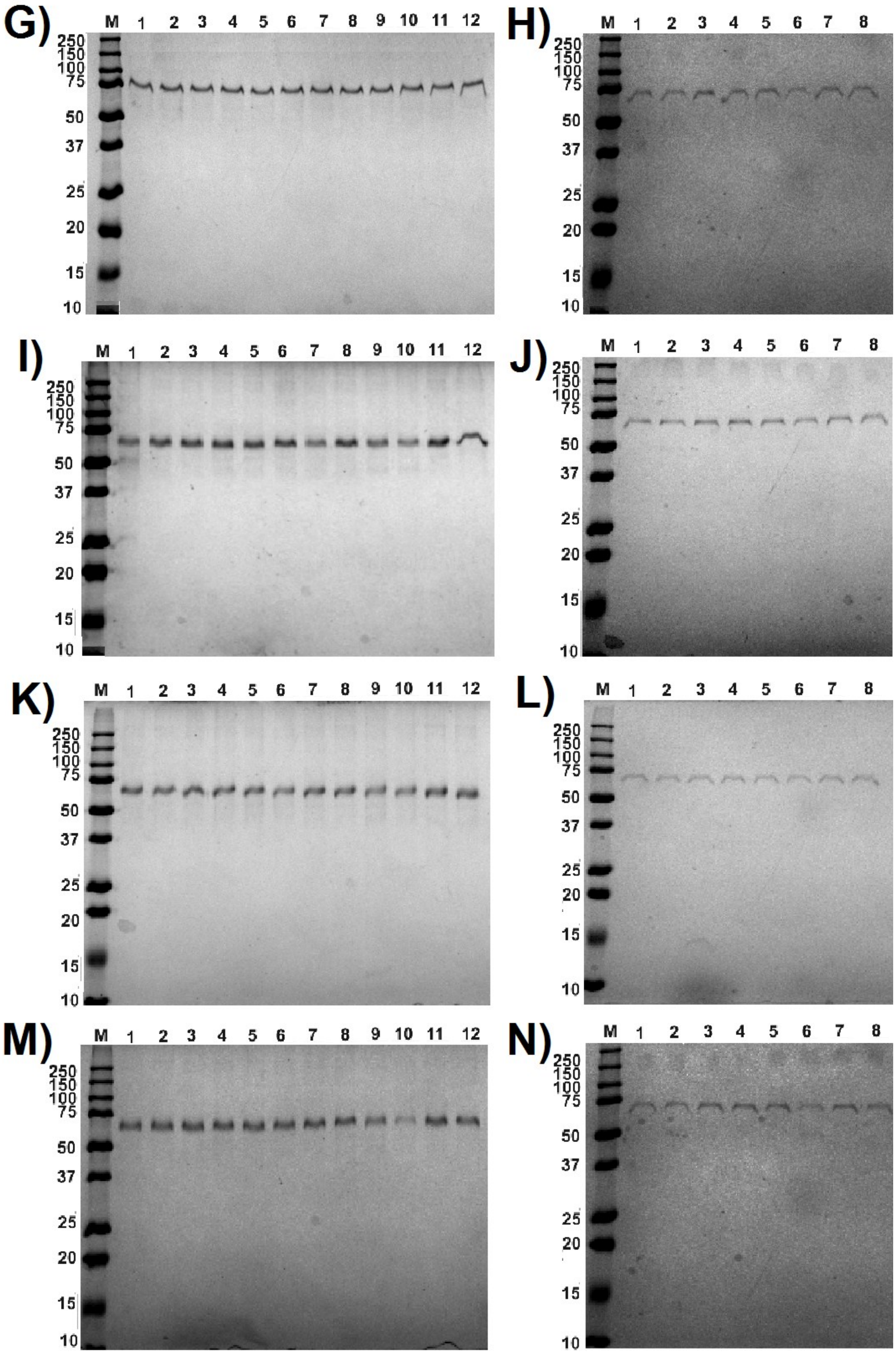

Figure S17: HSA with corresponding POM for 30 minutes at 20°C (1-4), at 37°C (5-8) and at 65°C (9-12) 1) 5 µg HSA, 2) 1:1 HSA:POM, 3) 1:10 HSA:POM, 4) 1:100 HSA:POM., 5) 5 µg HSA, 6) 1:1 HSA:POM, 7) 1:10 HSA:POM, 8) 1:100 HSA:POM, 9) 5 µg HSA, 10) 1:1 HSA:POM, 11) 1:10 HSA:POM, 12) 1:100 HSA:POM in NaOAc buffer [10 mM] 5.5 pH **A)** HSA with $[(Zn/W)_2\text{-}\beta\text{-}SbW_9]$ (**2**) after 30 min **B)** HSA with $[(Zn/W)_2\text{-}\beta\text{-}SbW_9]$ (**2**) after 3 days

C) HSA with [Mn-β-SbW_9] after 30min **D)** HSA with [Mn-β-SbW_9] after 3 days. **E)** HSA with [Cd_2Mn_2-β-SbW_9] (**3**) after 30 min. **F)** HSA with [β-$Cd_2Mn_2SbW_9$] (**3**) after 3 days.**G)** HSA with [$(Zn/W)_2$-β-SbW_9] (**2**) after 30 min **H)** HSA with [$(Zn/W)_2$-β-SbW_9] (**2**) after 3 days **I)** HSA with [Mn-β-SbW_9] after 30min **J)** HSA with [Mn-β-SbW_9] after 3 days. **K)** HSA with [Cd_2Mn_2-β-SbW_9] (**3**) after 30 min. **L)** HSA with [β-$Cd_2Mn_2SbW_9$] (**3**) after 3 days. **M)** HSA with $(TMA)_6[(Zn(H_2O))_6$(*B*-α-$SbW_9O_{33})_2$] (**1**) after 30 min. **N)** HSA with $(TMA)_6[(Zn(H_2O))_6$(*B*-α-$SbW_9O_{33})_2$] (**1**) after 3 days.

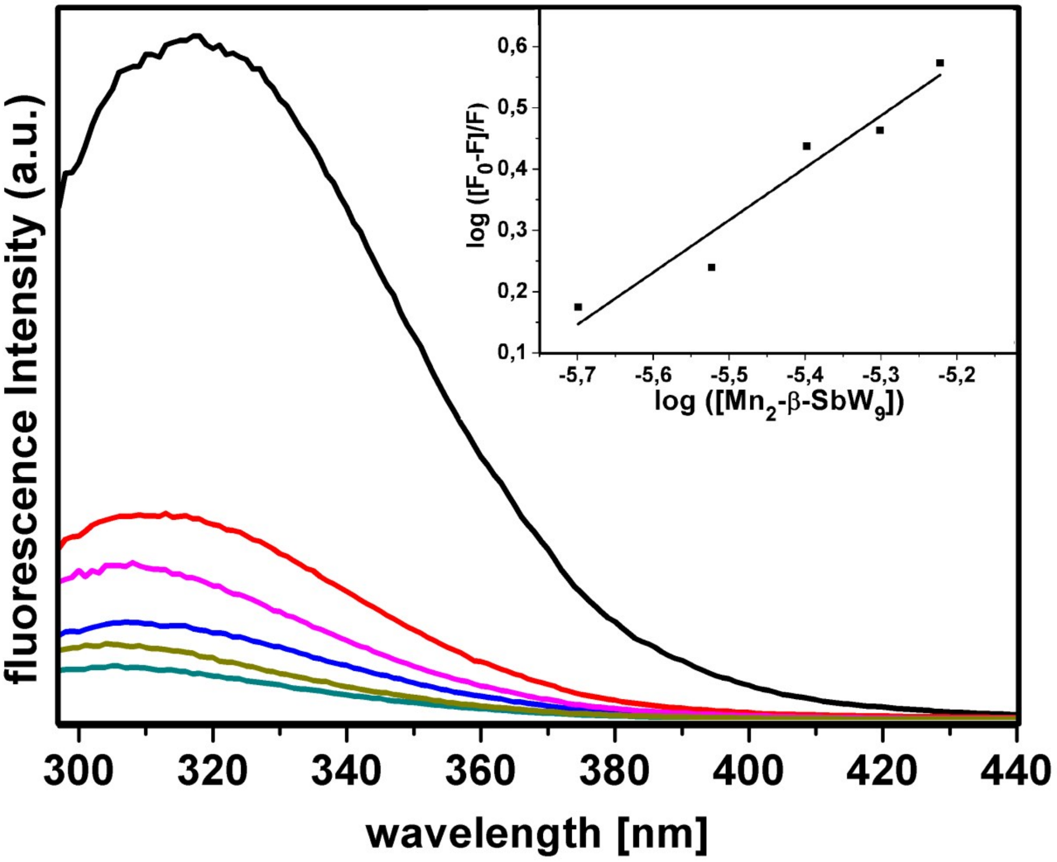

Figure S18: Emission fluorescence spectra of [Mn_2-β-SbW_9] with HSA [HSA] = 10^{-5} M^{-1}, in 10 mM NaOAc buffer pH 5.5. The top line is collected in the absence of [Mn_2-β-SbW_9] followed by stepwise increase (0.2, 0.3, 0.4, 0.5 and 0.6 fold of [Mn_2-β-SbW_9]). In the inset, the plot of the derived Stern–Volmer equation is depicted (with R^2 = 0.95).

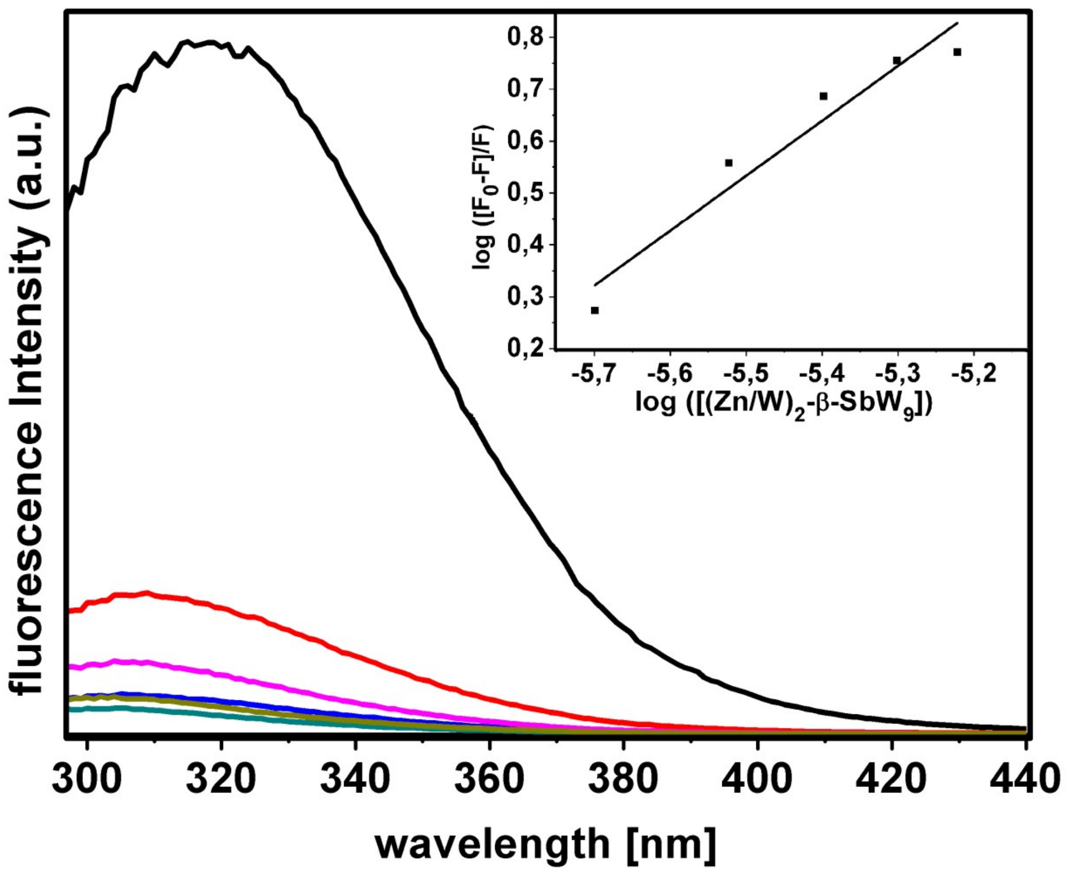

Figure S19: Emission fluorescence spectra of $[(Zn/W)_2\text{-}\beta\text{-}SbW_9]$ (**2**) with HSA [HSA] = 10^{-5} M^{-1}, in 10 mM NaOAc buffer pH 5.5. The top line is collected in the absence of $[(Zn/W)_2\text{-}\beta\text{-}SbW_9]$ (**2**) followed by stepwise increase (0.2, 0.3, 0.4, 0.5 and 0.6 fold of $[(Zn/W)_2\text{-}\beta\text{-}SbW_9]$ (**2**)). In the inset, the plot of the derived Stern–Volmer equation is depicted (with R^2 = 0.95).

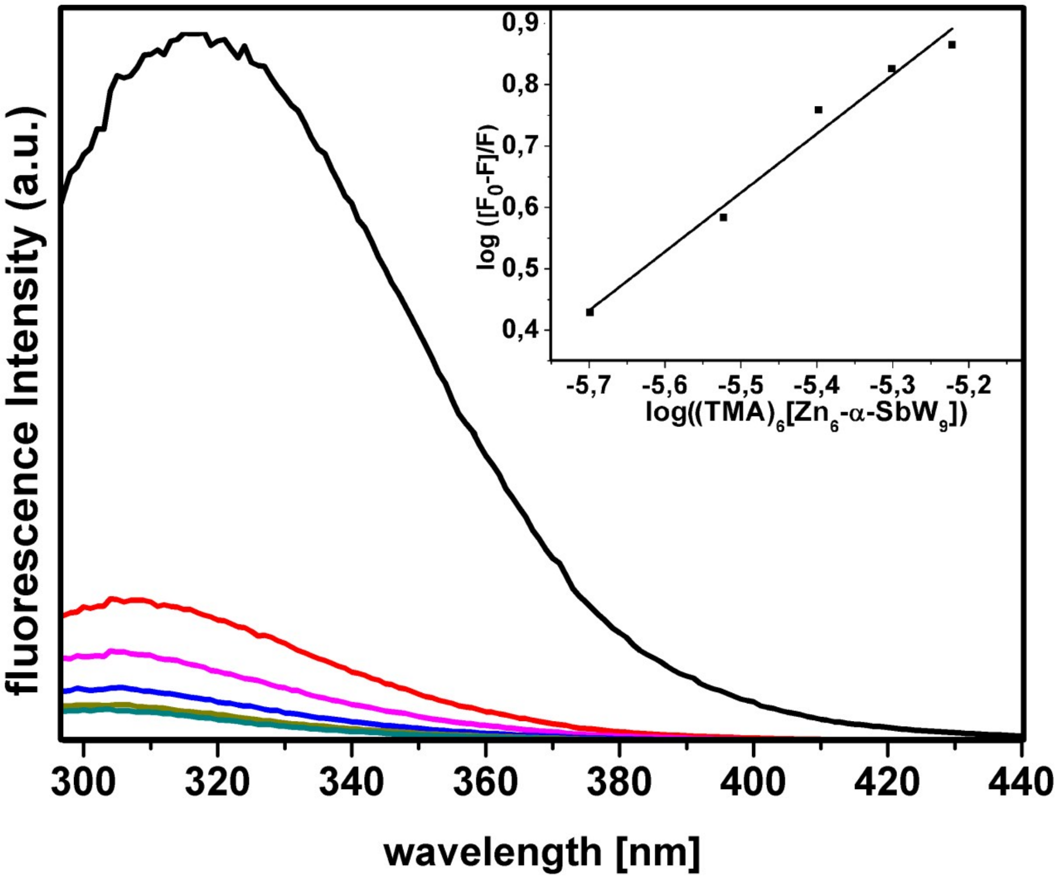

Figure S20: Emission fluorescence spectra of $(TMA)_6[(Zn(H_2O))_6(B\text{-}\alpha\text{-}SbW_9O_{33})_2]$ (**1**) with HSA [HSA] = 10^{-5} M^{-1}, in 10 mM NaOAc buffer pH 5.5. The top line is collected in the absence of $(TMA)_6[(Zn(H_2O))_6(B\text{-}\alpha\text{-}SbW_9O_{33})_2]$ (**1**) followed by stepwise increase (0.2, 0.3, 0.4, 0.5 and 0.6 fold of $(TMA)_6[(Zn(H_2O))_6(B\text{-}\alpha\text{-}SbW_9O_{33})_2]$ (**1**). In the inset, the plot of the derived Stern–Volmer equation is depicted (with R^2 = 0.98).

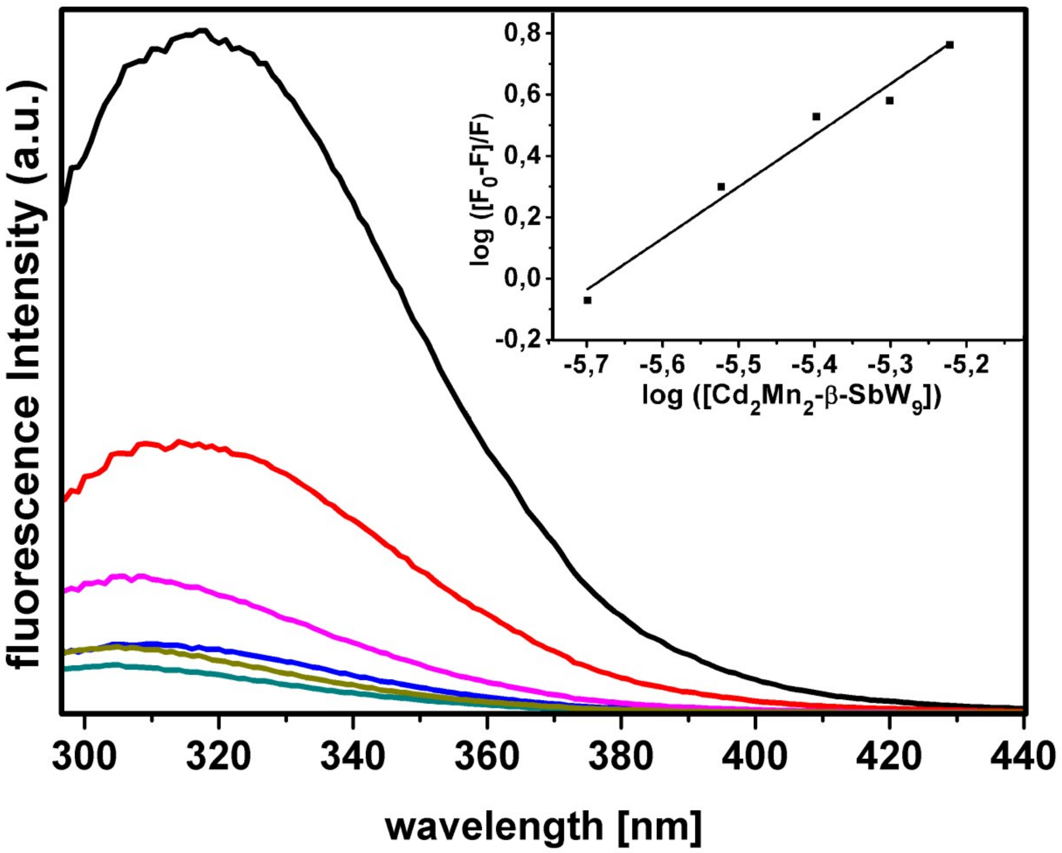

Figure S21: Emission fluorescence spectra of [Cd_2Mn_2-β-SbW_9] (**3**) with HSA [HSA] = 10^{-5} M^{-1}, in 10 mM NaOAc buffer pH 5.5. The top line is collected in the absence of [Cd_2Mn_2-β-SbW_9] (**3**) followed by stepwise increase (0.2, 0.3, 0.4, 0.5 and 0.6 fold of [Cd_2Mn_2-β-SbW_9] (**3**)). In the inset, the plot of the derived Stern–Volmer equation is depicted (with R^2 = 0.98).

Table S18: Quenching constants K_q and number (n) of binding molecules for the HSA protein at pH 5.5

POM	K_q [M^{-1}]	n
**[Mn-β-SbW_9]**	**1.1 × 10^5**	**0.85**
**[$(Zn/W)_2$-β-SbW_9]** (**2**)	**2.2 × 10^6**	**1.10**
**$(TMA)_6[(Zn(H_2O))_6$(*B*-α-$SbW_9O_{33})_2$]** (**1**)	**0.8 × 10^6**	**0.96**
**[Cd_2Mn_2-β-SbW_9]** (**3**)	**3.4 × 10^9**	**1.60**

10 References

[1] M. Bösing, I. Loose, H. Pohlmann, B. Krebs, *Chem. Eur. J.* 1997, **3**, 1232.
[2] U.K. Laemmli, Nature 1970, **227**, 680-685.
[3] J. Chen, G. Song, Y. He, Q. Yan, *Microchim. Acta*, 2007, **159**, 79-85.
[4] Bruker SAINT v7.68A Copyright © 2005-2016 Bruker AXS
[5] O. V. Dolomanov, L. J. Bourhis, R. J. Gildea, J. A. K. Howard, H. Puschmann, *J. Appl. Cryst.* 2009, **42**, 339-341.
[6] C. B. Huebschle, G. M. Sheldrick, B. Dittrich, *J. Appl. Cryst*. 2011, **44**, 1281-1284.
[7] G. M. Sheldrick, 1996. SHELXS. University of Göttingen, Germany.
[8] G. M. Sheldrick, 1996. SHELXL. University of Göttingen, Germany.
